# Tailoring Interface Energies via Phosphonic Acids to Grow and Stabilize Cubic FAPbI_3_ Deposited by Thermal Evaporation

**DOI:** 10.1021/jacs.4c03911

**Published:** 2024-06-27

**Authors:** Andrés-Felipe Castro-Méndez, Farzaneh Jahanbakhshi, Diana K. LaFollette, Benjamin J. Lawrie, Ruipeng Li, Carlo A. R. Perini, Andrew M. Rappe, Juan-Pablo Correa-Baena

**Affiliations:** † School of Materials Science and Engineering, Georgia Institute of Technology, North Ave NW, Atlanta, Georgia 30332, United States; ‡ Department of Chemistry, University of Pennsylvania, Philadelphia, Pennsylvania 19104-6323, United States; § The Center for Nanophase Materials Sciences, Oak Ridge National Laboratory, Oak Ridge, Tennessee 37831, United States; ∥ Materials Science and Technology Division, Oak Ridge National Laboratory, Oak Ridge, Tennessee 37831, United States; ⊥ National Synchrotron Light Source II (NSLS-II), Brookhaven National Laboratory, Upton, New York 11967, United States; # School of Chemistry and Biochemistry, Georgia Institute of Technology, North Ave NW, Atlanta, Georgia 30332, United States

## Abstract

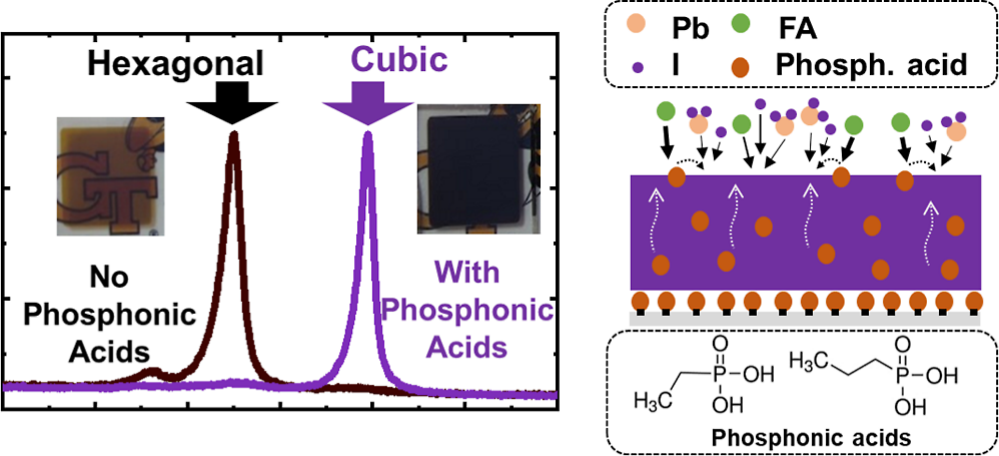

Coevaporation of formamidinium lead iodide (FAPbI_3_) is a promising route for the fabrication of highly efficient and scalable optoelectronic devices, such as perovskite solar cells. However, it poses experimental challenges in achieving stoichiometric FAPbI_3_ films with a cubic structure (α-FAPbI_3_). In this work, we show that undesired hexagonal phases of both PbI_2_ and FAPbI_3_ form during thermal evaporation, including the well-known 2H-FAPbI_3_, which are detrimental for optoelectronic performance. We demonstrate the growth of α-FAPbI_3_ at room temperature via thermal evaporation by depositing phosphonic acids (PAc) on substrates and subsequently coevaporating PbI_2_ and formamidinium iodide. We use density-functional theory to develop a theoretical model to understand the relative growth energetics of the α and 2H phases of FAPbI_3_ for different molecular interactions. Experiments and theory show that the presence of PAc molecules stabilizes the formation of α-FAPbI_3_ in thin films when excess molecules are available to migrate during growth. This migration of molecules facilitates the continued presence of adsorbed organic precursors at the free surface throughout the evaporation, which lowers the growth energy of the α-FAPbI_3_ phase. Our theoretical analyses of PAc molecule–molecule interactions show that ligands can form hydrogen bonding to reduce the migration rate of the molecules through the deposited film, limiting the effects on the crystal structure stabilization. Our results also show that the phase stabilization with molecules that migrate is long-lasting and resistant to moist air. These findings enable reliable formation and processing of α-FAPbI_3_ films via vapor deposition.

## Introduction

Despite the rapid increase in power conversion efficiency in perovskite solar cells (PSCs)^1−3^ in academic laboratories, the scalability of this technology remains a significant challenge for its commercialization. PSCs are primarily fabricated using solution processes that have limited control over thickness and uniformity over large substrates and that impose constraints on the choice of layer materials due to solvent orthogonality requirements.^4,5^ Vapor deposition has the potential to overcome these challenges. The thickness and uniformity of vapor-deposited films can be easily controlled with the use of quartz crystal microbalances (QCMs) and by tuning the distance between the source and the substrate. Likewise, the lack of solvents facilitates the deposition of multiple layers without causing damage to the rest of the device, enhancing the overall device stability.^6^ Despite these advantages, thermal evaporation of organic–inorganic hybrid materials suffers from a lack of control of the stoichiometry due to low sticking coefficients of the organic cations to QCMs^7,8^ and the thermal degradation of the organics,^9,10^ which lead to the formation of secondary phases.^11^ In this context, formamidinium iodide (FAI) has demonstrated better deposition control than the widely studied methylammonium iodide (MAI), as FAI exhibits a higher sticking coefficient to the QCMs and better thermal stability. Furthermore, the main FAI byproducts in the evaporation process, hydrogen cyanide and *sym*-triazine, have not been shown to incorporate in the deposited materials.^9,10,12^

Among the many metal halide perovskite (MHP) compositions, FAPbI_3_ is one of the most promising candidates for solar cell applications. The α-FAPbI_3_ phase (the perovskite phase with the space group *Pm*3̅*m*) has a band gap of 1.48 eV, close to the Shockley–Queisser maximum efficiency for single junction solar cells.^13,14^ However, combining FAPbI_3_ and PbI_2_ can form different polytypes of the hexagonal FAPbI_3_ phase that are named with the Ramsdell notation as the 2H, 4H, and 6H. The 2H phase is also termed the δ phase and only contains face-sharing octahedra in its structure, whereas 4H and 6H, because of stacking faults, contain a mixture of both corner and face-sharing octahedra.^15^ While these hexagonal phases are often identified in the literature for FAPI_3_, PbI_2_ without the A-site cation^16^ can also form these hexagonal phases. The presence of hexagonal phases limits the use of pure FAPbI_3_ as the absorber layer in PSCs^17,18^ due to its indirect and large band gap of around 2.4 eV. Moreover, studies have shown that once the α-FAPbI_3_ perovskite is formed at high temperatures, it is stable at room temperature in an inert environment.^19^

Accordingly, many strategies to foster the formation of α-FAPbI_3_ at ambient conditions have been utilized. Alloying FAPbI_3_ with Cs^+^ and MA^+^ is an effective way to favor the α phase formation, via both solution and evaporation.^20−24^ However, it has been shown that MA^+^ causes instabilities in solar cells, mainly due to the fragmentation of MA molecules into H, CH_2_, CH_3_, CH_4_, NH_2_, NH_3_, and CH_3_NH_2_ under thermal stress.^25^ Moreover, hydrogen migration has also been linked to the formation of defects that affect the device performance.^26^ Likewise, Cs^+^ can migrate and segregate, leading to the formation of CsPbI_3_ nonperovskite phases.^27^ Previous research on thermal evaporation of MHPs has shown that the substrate plays a crucial role in the growth mechanism of the MHP layer. Studies on MAPbI_3_ demonstrate that the thickness of the deposited layer is dependent on the substrate chemistry, with the sticking factor of MAI being more strongly affected by the substrate.^28^ Likewise, Roß et al.^29^ showed that α-FAPbI_3_ grows at room temperature when both an excess of FAI is present and when the substrate is treated with [2-(3,6-dimethoxy-9*H*-carbazol-9-yl)ethyl]phosphonic acid (MeO-2PACz). Recently, Feeney et al. showed that this effect is also beneficial in the evaporation of Cs_0.13_FA_0.87_Pb(I_0.95_Cl_0.05_)_3_, where the presence of phosphonic acids (and presumably of Cs) increases the activation energy for the transition from the cubic to the hexagonal phase.^30^ However, the influence of the phosphonic acid molecules beyond the interface and how this relates to the formation of the α-FAPbI_3_ remains unexplored.

In this study, we deposit PAc molecules on FTO substrates to investigate the role of surface chemistry on the phase purity and composition of coevaporated FAPbI_3_ films. To understand the growth mechanisms of the α phase, we select a range of PAc molecules with different additional moieties that can interact both with the MHP and with one another in varying degrees. These molecules are deposited by spin coating on the substrates where FAPbI_3_ precursors are thermally evaporated. Performing X-ray photoelectron spectroscopy (XPS), we show that FA^+^-deficient films are formed on untreated substrates, leading to the formation of the hexagonal phases, including the δ-FAPbI_3_ phase. Interestingly, when the surface is treated with some PAc molecules, we measure a higher incorporation of the organic precursors into the film, and the α-FAPbI_3_ is induced as the dominant crystalline phase. We performed density functional theory (DFT) calculations to compare the stability of thin films of α and δ FAPbI_3_ perovskites on the fluorine-doped tin oxide (FTO) substrate in the presence and absence of a PAc molecule. These calculations enable us to develop a theoretical model that relates the growth energy to the grown thickness for each MHP phase, explaining how surface treatments with PAc molecules can help foster the growth of α-FAPbI_3_. We further calculate the binding energies (BEs) between the substrate and the PAc as well as between the PAc molecules. This allows us to understand how likely it is for PAc molecules to migrate through the perovskite layer if deposited in amounts exceeding that required to form a monolayer on the substrate. Our simulations and experiments reveal that the migration of PAc molecules through the MHP film is essential for promoting the growth of α-FAPbI_3_. This process is enabled by a consistently high sorption rate of FAI throughout the entire evaporation process and by the reduction of the growth energy of α-FAPbI_3_ compared to the δ phase in the presence of PAc molecules. We propose that phosphonic acids with weaker ligand–ligand binding, such as propylphosphonic acid (PPAc), are more likely to migrate and thus contribute to the growth of α-FAPbI_3_ films as they are deposited layer by layer in thermal evaporation.

## Results and Discussion

### Thermal Coevaporation of FAPbI_3_ on Pristine Substrates

**Figure 1:**
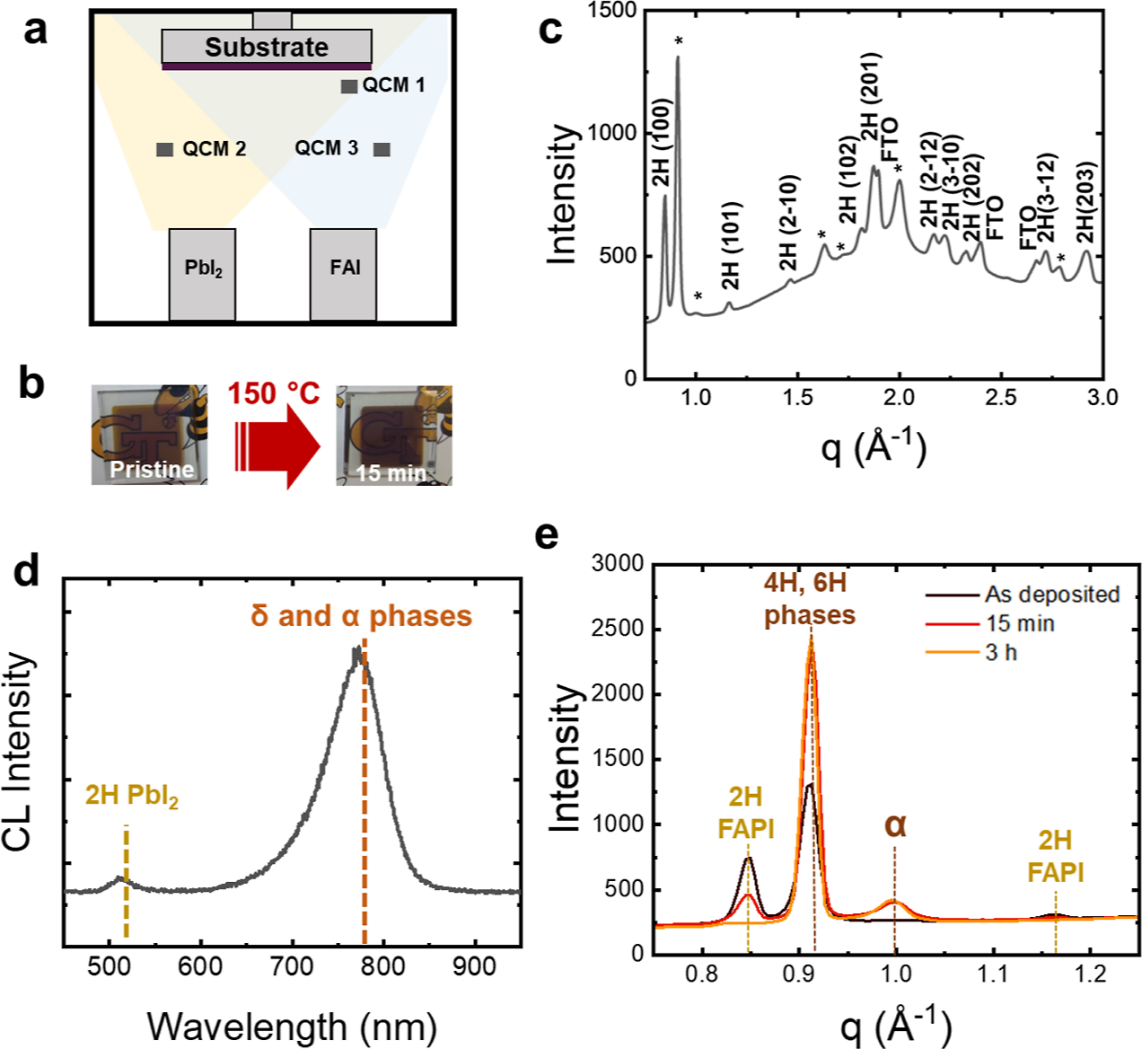
Growth of PbI_2_ and FAPI_3_ hexagonal phases on FTO. (a) Schematic of the evaporation process. (b) Photographic documentation of the evolution of the films with the annealing time at 150 °C. (c) Circular integration of GIWAXS pattern of the as-deposited FAPbI_3_ film. The 2H labels belong to FAPI_3_ and * indicates the position of XRD peaks incompatible with the δ-FAPI_3_ phase and suggests the presence of other hexagonal polytypes from both PbI_2_ and FAPI_3_. (d) Cathodoluminescence spectrum of the as-deposited FAPbI_3_ film. (e) Evolution of the circular integration of GIWAXS patterns FAPbI_3_ films with annealing time at 150 °C.

This study uses coevaporation of PbI_2_ and FAI to deposit FAPbI_3_ films, as shown in Figure 1a. Three QCMs, denoted as QCM 1, QCM 2, and QCM 3 in Figure 1a, are used to monitor the total thickness of the deposited layer at the substrate, to control the deposition rate of PbI_2_, and that of FAI, respectively. To ensure the appropriate ratio between the deposition rates of PbI_2_ and FAI, the QCMs are calibrated as indicated in the Experimental Section. Upon deposition of FAPbI_3_, the deposited film exhibits a yellow/light brown color, suggesting the presence of large amounts of hexagonal phases (Figure 1b). To identify the crystalline phases formed in the deposited film, we conducted grazing incidence wide-angle X-ray scattering (GIWAXS) of the FAPbI_3_ film deposited on FTO (Figure 1c). The complete 2D GIWAXS pattern of the film is reported in Figure S1, while in Figure 1c, we show the circular integration of the pattern, which offers easier identification of the peak positions. The diffraction pattern reveals a characteristic peak of the δ-FAPbI_3_ phase (predominant peak expected at *q* = 0.84 Å^–1^)^31^ and almost complete absence of crystalline α-FAPbI_3_ (predominant peak expected at *q* = 0.99 Å^–1^).^31^ Notably, the most intense diffraction peak is at *q* = 0.91 Å^–1^, matching with the diffraction expected from the 2H phase of PbI_2_.^16^ However, two peaks at *q* = 1.63 Å^–1^ and *q* = 1.73 Å^–1^ are also present in Figure 1c and cannot be attributed to the 2H phase of PbI_2_, to the α-FAPbI_3_, or the δ-FAPbI_3_. Although these peaks are close to the diffraction peaks of the 4H and 6H phases for FAPbI_3_ (Figure S2), the peaks match with those expected from the 6H phase of PbI_2_ (ICSD 024265), suggesting that this phase is formed when MHP thin films are deposited via vapor.^16^ To ensure that the phases observed in GIWAXS represent the as-deposited films and are not a consequence of sample degradation during transportation to the beamline facility, we conducted X-ray diffraction (XRD) of a thin film measured immediately after deposition (Figure S3), and the phases observed are consistent with those detected with GIWAXS.

To corroborate our understanding of the secondary phases present in the film, we measured cathodoluminescence (CL), as shown in Figure 1d. CL detects the radiation emitted by the sample upon excitation with an electron beam. This technique proves useful for detecting and mapping distinct phases within the sample, as the wavelength of the emission depends on the bandgap of the material.^32^ The FAI–PbI_2_ coevaporated samples show homogeneous distribution of the CL map (Figure S4). The spectra exhibit a weak emission peak centered at a wavelength of 505 nm, matching the emission of 2H PbI_2_.^32^ A broader and more intense peak is observed centered at an emission wavelength of 790 nm, blue-shifted with respect to the emission peak of pure α-FAPbI_3_ (Figure 1c).^32,33^ This peak is also asymmetric, suggesting the presence of more than one phase. Such shift and broadening of the emission has also been observed in the PL data of MHP films and can be attributed to hexagonal phases of FAPbI_3_ or possibly from the 6H of PbI_2_.^15^ In addition, the observed blue shift in the emission peak is consistent with theoretical predictions of the band gap for the hexagonal polytypes.^34^ A mixture of hexagonal polytypes is therefore likely to be present in the film.

A common approach for converting δ-FAPbI_3_ films into the α-FAPbI_3_ phase in solution-processed films is to thermally anneal them at 150 °C, the temperature at which the α phase is thermodynamically more stable compared to its hexagonal counterparts.^18^Figure 1e shows the circularly integrated GIWAXS patterns (Figure S5 shows the XRD data) as a function of annealing time at 150 °C. Annealing leads to the decrease of the δ-FAPbI_3_ (*q* = 0.84 Å^–1^, 2θ = 11.65°) and to the emergence of a small peak that belongs to the α-FAPbI_3_ (*q* = 0.99 Å^–1^, 2θ = 13.9°). Moreover, the peak at *q* = 0.91 Å^–1^ (2θ = 12.5°, which belongs to the hexagonal polymorphs) increases in intensity. In solution processing, this annealing approach has been reported to suppress all nonperovskite phases and promote the growth of the α-FAPbI_3_ (*q* = 0.99 Å^–1^), which is not the case for our evaporated films. Furthermore, annealing of the films leads to an increase in the intensity of the absorption shoulders at 500 and 560 nm observed by UV–vis spectroscopy in Figure S6, which are typically related to the δ-FAPbI_3_ phase.^35^

### Thermal Coevaporation of FAPbI_3_ on PAc-Treated Substrates

**Figure 2:**
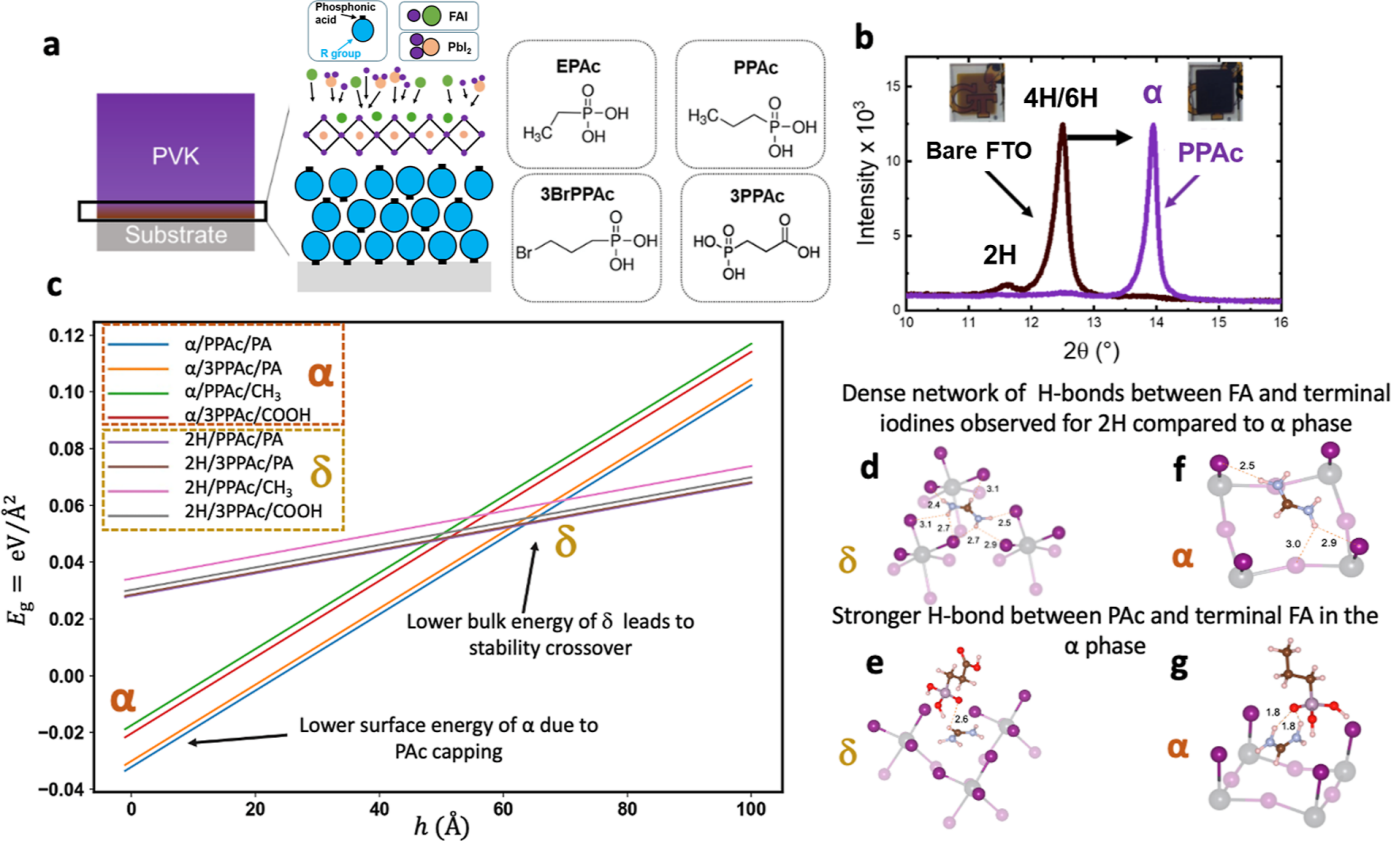
Growth mechanisms of α-FAPbI_3_ on substrates treated with PPAc molecules. (a) Schematics of the substrate treatment with select phosphonic acids. (b) Comparison of the XRD patterns of FAPbI_3_ films on substrates with and without PPAc (3 mM in ethanol) treatment. (c) Model of the growth energies as a function of FAPbI_3_ layer thickness (*h*) with PPAc and 3PPAc molecules. A denser network of stronger H-bonds between FA and terminal iodines in 2H is shown in (d) compared to α phase (f). The interactions between the PAc and terminal FA in the 2H-FAPbI_3_phase (e) are weaker than in the α-FAPbI_3_ phase (g), which show stronger hydrogen bond formation (H···O = 1.8 Å). All bond lengths are given in Å with the threshold of 3.1 Å.

To promote the growth of α-FAPbI_3_ without the need for postannealing steps or compositional engineering of the MHP, we deposited the FTO substrate with PAc molecules, as shown in the schematic provided in Figure 2a. This treatment aims to stabilize the α-FAPbI_3_ by creating molecular interactions (e.g., hydrogen bonds) between the PAc molecule and the polar MHP precursors during evaporation. We selected a variety of PAc molecules with additional moieties capable of interacting with both the MHP precursor and with each other to varying degrees. We considered PAc molecules with aliphatic moieties such as ethyl-phosphonic acid (EPAc) and PPAc, PAc molecules with halide moieties such as 3-bromopropylphosphonic acid (3BrPPAc), with moieties that can form hydrogen bonding such as 3-phosphonopropionic acid (3PPAc), and with moieties containing π orbitals such as phenyl phosphonic acid (PhPAc). Figure S7 shows the XRD of the MHP films deposited on substrates treated with these molecules. We observed that PAc molecules with ligands that interact weakly with each other, such as those in PPAc and EPAc, are particularly effective in promoting the growth of α-FAPbI_3_. PPAc and EPAc are the only molecules we studied as surface modifiers that lead to the formation of mostly α-FAPbI_3_, with a peak at 2θ = 13.95°. In contrast, molecules with ligands that tend to interact more strongly with each other, such as halogens, oxygens, and phenyl rings, lead to the formation of films containing both α and hexagonal phases of PbI_2_ and FAPbI_3_. For instance, the use of 3BrPPAc induces a mixture of α- and hexagonal polytypes of PbI_2_ and FAPbI_3_, while 3PPAc results in films composed solely of hexagonal polytypes of PbI_2_ and FAPbI_3_. These results suggest that the characteristics of the ligands, and not just the PAc group, play a key role in facilitating the growth of α-FAPbI_3_.

We performed first-principles studies (Figure 2c–g) to shed light on the mechanism by which certain ligands in the PAc molecules promote the growth of α-FAPbI_3_, as experimentally observed in Figure 2b. We modeled the growth of FAPbI_3_ on the FTO substrate treated by two different PAc molecules (PPAc and 3PPAc) that had exhibited contrasting results in our experiments. As described earlier, we observed, experimentally, the formation of α-FAPbI_3_ upon coating the substrate with PPAc, while the 3PPAc treatment formed hexagonal phases. To unravel the underlying mechanisms, we employed quantum mechanical models to simulate the growth of FAPbI_3_ slabs interacting with PPAc or 3PPAc. We considered the capping modes involving the phosphonate (PA), methyl (CH_3_ group of PPAc), and carboxyl (COOH group of 3PPAc) ligands, on both FAI- and PbI_2_-terminated slabs of FAPbI_3_ and calculated their corresponding growth energies with DFT. Figures S8–S10 show all the capping modes and their corresponding growth energies for both the δ- and α-FAPbI_3_ phases as a function of the grown thickness. By calculating the energies of capped films of varying thicknesses, we deduced the capping energy (surface energy) and the bulk energy for each PAc and interface type (see Table 1). More specifically, Figure 2c demonstrates the energetic competition between these phases as a function of modeled layer thickness. The results show that the lowest growth energy is achieved upon capping via the PA group for both PPAc and 3PPAc. This is consistent with the Pb–O distance of 2.4 Å, indicating the formation of covalent bonds for PA-capping modes (of the Pb-terminated slabs) as well as H-bond formation between PAc and terminal FA (of FAI-terminated slabs as shown in Figure 2d–g). Such bond formations result in higher stabilization due to favorable ligand capping, and thus lowering the growth energy. Table 1 summarizes the calculated energetics per eqs S1–S3 required to obtain the growth energy per eq S4. Most notably, the tabulated data suggest that the capping energies are in general higher (less favorable) for both FAI and PbI_2_ terminations of δ and remain generally comparable between the two terminations (FAI and PbI_2_) for each phase. To rationalize this key observation, we studied the molecular surface chemistry of both phases before and after PAc molecule capping. As displayed in Figure 2 (d–g), a denser network of stronger H-bonds between FA^+^ ions and terminal iodines is formed in the case of 2H as compared to the α phase (in line with the lower bulk energy of delta-FAPbI_3_). This weaker H-bonding within the α-FAPbI_3_ phase explains the formation of shorter, stronger hydrogen bonds (H···O = 1.8 Å) between PAc and terminal FA in the α- (Figure 2g) in comparison with the δ-FAPbI_3_ phase (Figure 2e) upon capping. Our calculations also showed that capping energies of the COOH mode are lower (more negative) in the FAI termination compared to the PbI_2_ termination due to H-bond formation between the carboxylic group of the molecule and the terminal FA (Figure S8, d and h). The α phase continues to grow until it reaches a certain thickness where the bulk formation energy contribution becomes large enough to cause a crossover in the growth energy plot (Figures 2c and S10). Our model shows that all the interations of both PPAc and 3PPAc with the FA can help promote the growth of the α-FAPbI_3_ phase. However, as reported in Table 1, the energy corresponding to capping via the PA group is significantly more favorable than the CH_3_ and COOH modes for both α and δ phases of FAPbI_3_.

**Table 1:** FAPbI_3_ Bulk Formation Energy (*E*_form_), Bare Separation Energy (*E*_sep_), and Capping Energy (*E*_cap_) Calculated per eqs S1–S3, Corresponding to FAI-and PbI_2_-Terminated Slab Models of α- and 2Η-FAPbI_3_^a^

capping molecule	PPA-capped		3PPA-capped	
mode, grown phase	via PA α, 2H	via CH_3_ α, 2H	via PA α, 2H	via COOH α, 2H
PbI_2_ Termination				
*E*_form_ (meV/Å^3^)	1.34, 0.39	1.34, 0.39	1.34, 0.39	1.34, 0.39
*E*_sep_ (meV/Å^2^)	2.21, 39.18	2.21, 39.18	2.21, 39.18	2.21, 39.18
*E*_cap_ (meV/Å^2^)	–36.98, −13.02	–13.74, −4.41	–36.37, −11.90	–26.11, −7.20
FAI Termination				
*E*_form_ (meV/Å^3^)	1.34, 0.39	1.34, 0.39	1.34, 0.39	1.34, 0.39
*E*_sep_ (meV/Å^2^)	2.21, 39.18	2.21, 39.18	2.21, 39.18	2.21, 39.18
*E*_cap_ (meV/Å^2^)	–34.40, −11.08	–19.74, −5.00	–32.33, −10.69	–22.59, −8.97

a The energy of growing FAPbI_3_ (*E*_g_) at a thickness of *h* (Figures 2c and S10) composed of these terms can be obtained via eq S4.

**Figure 3:**
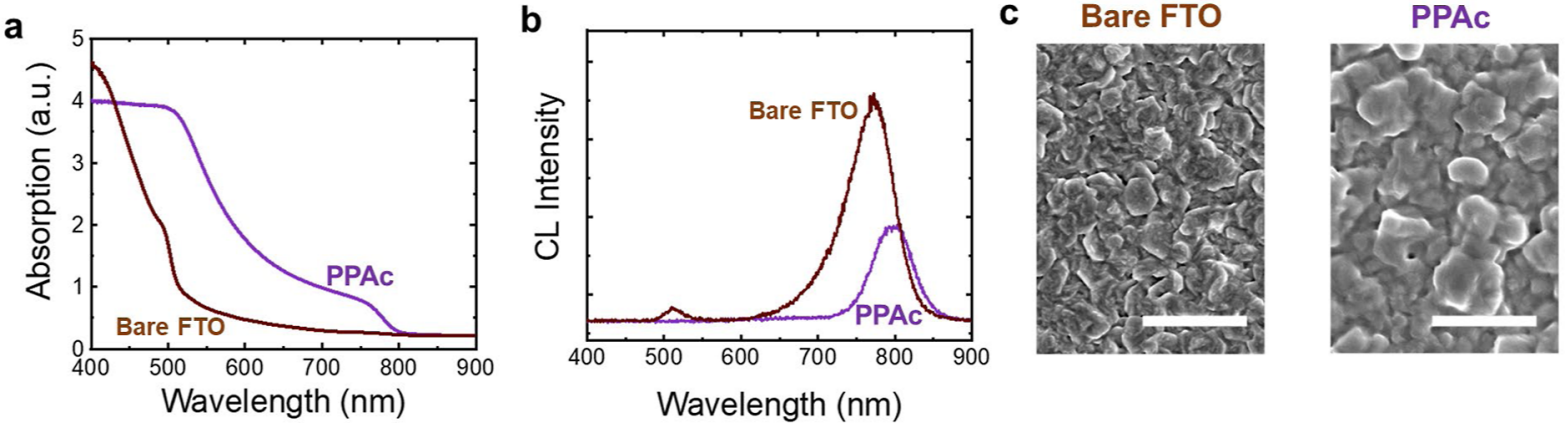
Optical properties and morphology assessment of thin films. (a) UV–vis absorption spectra, (b) CL spectra, and (c) SEM images of FAPbI_3_ films on substrates with and without PPAc treatment. Scale bar is 1 μm.

We investigated the effects of structural changes induced by PPAc molecules on the properties and morphology of thin films as shown in Figure 3. Figure 3a compares the UV–vis absorption spectra of FAPbI_3_ films fabricated on bare and PPAc-treated FTO substrates. The films deposited on PPAc-treated substrates exhibit a characteristic absorption edge at around 800 nm, which is close to the absorption edge of the α-FAPbI_3_ phase. Likewise, the CL spectra in Figures 3b and S11 show narrow peaks centered at around 800 nm and wide peaks centered around 760 nm for the PPAc-treated samples and those deposited on bare FTO, respectively. These results further confirm the formation of the α-FAPbI_3_ for the PPAc-treated samples. The PPAc treatment has strong effects on the morphology of the thin films. Scanning electron microscopy (SEM) images show the formation of larger grains for samples prepared on the PPAc-treated substrate when compared to the bare FTO samples, as shown in Figure 3c, suggesting that PPAc treatment has a strong effect on the sorption of the co-evaporated precursors.

**Figure 4:**
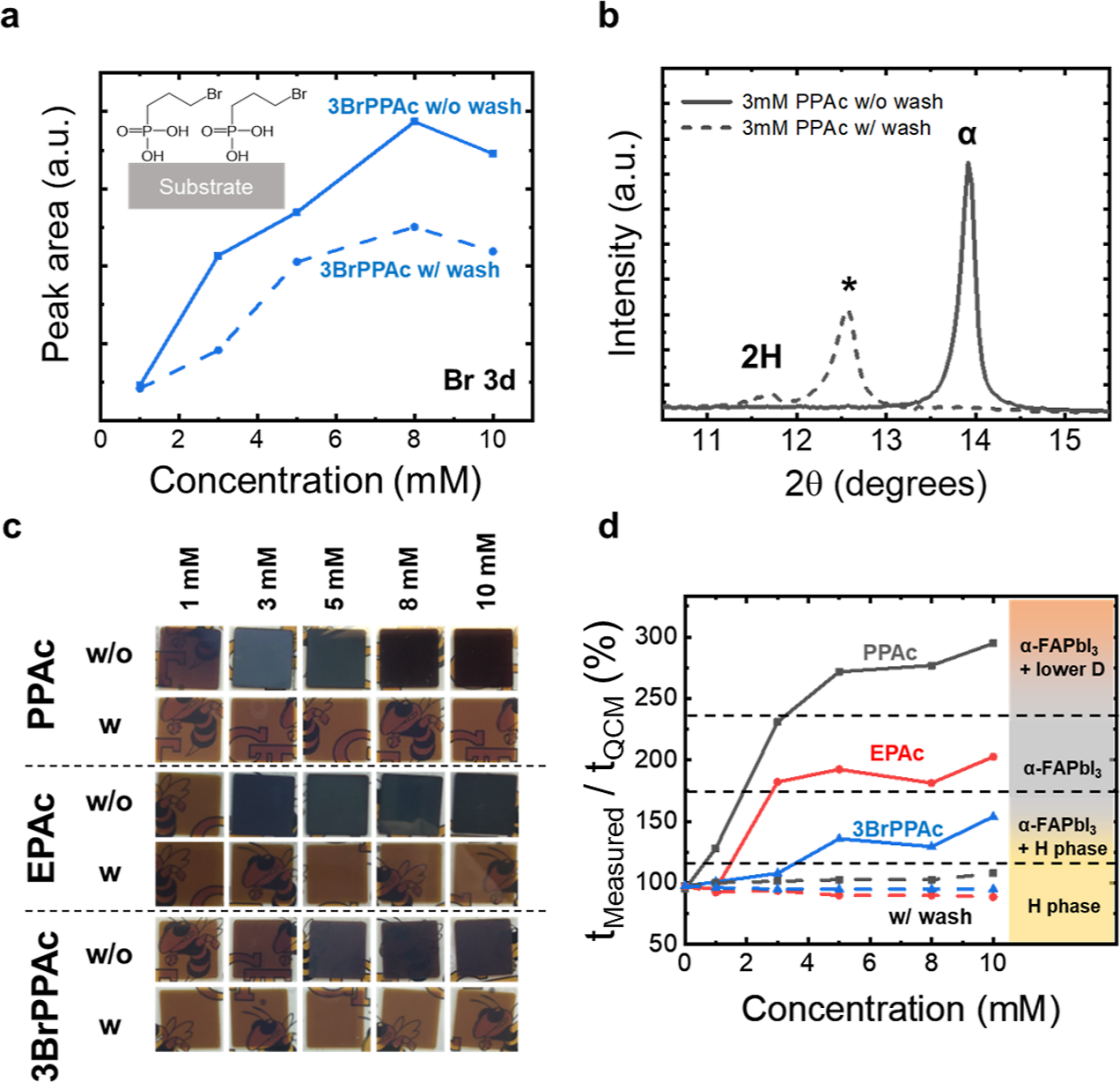
Effect of concentration of PAc solution and ethanol wash on the phase purity. (a) Br 3d peak area of 3BrPPAc-treated substrates with and without ethanol wash as measured by XPS. (b) Comparison of XRD patterns of films deposited on PPAc-treated substrates with and without ethanol wash. (c) Pictures of FAPbI_3_ films deposited on substrates treated with varying concentrations and PAc molecules. (d) Ratio of measured thickness (*t*_Measured_) on substrates to the predicted thickness by the QCM (*t*_QCM_) for FAPbI_3_ films deposited on substrates treated with varying concentrations and PAc molecules.

To experimentally separate the effects of the ligand from the PA group, and to unravel the impact of excess phosphonic acid molecules on the formation of the α-FAPbI_3_, we removed excess PAc molecules by spin coating ethanol on the treated substrates (hereafter referred to as ethanol wash). This procedure ensures the removal of excess PAc molecules not directly adsorbed to the FTO substrate that could interact with the perovskite layer with random orientations. We believe that this approach maintains close to a single monolayer of molecules, such that the ligand is accessible to the growing FAPbI_3_, while the PA group remains buried and bound to the FTO. We measured XPS to confirm the removal of excess PAc molecules from the surface. We used 3BrPPAc as a case study (shown in Figure 4a) and measured the Br 3d XPS peak area on the substrates treated with varying concentrations of the 3BrPPAc solution, before and after the ethanol wash. The Br 3d peak area increases with concentration in solution of the 3BrPPAc, when no wash is performed. Instead, when the concentration of 3BrPPAc is higher than 5 mM, there is no change in the Br 3d peak area after the ethanol wash, suggesting that PAc concentrations below 5 mM may lead to incomplete coverage of the substrate. In contrast, complete coverage can be achieved at PAc concentrations above 5 mM, with the removal of the unbound (excess) 3BrPPAc during the ethanol wash leaving a monolayer of PAc molecules on the substrate after the wash. In all cases of using ethanol-washed substrates, we observe no evidence of α-FAPbI_3_ phase stabilization (Figure S7). Instead, we grow thin films composed of a mixture of hexagonal phases, nearly identical to the cases with no PAc treatment (Figure 4b,c). As such, the removal of the excess PAc molecules revealed their key role in the α phase formation. These findings suggest that a single monolayer of PAc molecules is insufficient for the growth of α-FAPbI_3_, in agreement with the DFT calculations that predict the α phase formation is most promoted by the PA group capping mode and that the formation of α-FAPbI_3_ is contingent upon the excess phosphonic acid molecules.

Analogous to the effect of ethanol wash, a solution of PAc molecules at low concentrations also prevents the formation of the α-FAPbI_3_ phase. In the photographs shown in Figure 4c, higher concentrations of PAc molecules lead to thin films with darker color, suggesting the formation of the α-FAPbI_3_. This effect can be clearly seen in the films treated with 3BrPPAc (Figure S12). Substrates treated with a solution of 1 mM of 3BrPPAc show that the film is composed solely of hexagonal phases. However, as the concentration of 3BrPPAc increases, the content of the α phase also increases, achieving an α to hexagonal peak intensity ratio of 2.8:1 at a concentration of 10 mM (Figure S12). Similar trends are observed for all PAc molecules tested as can be seen in the XRD patterns in Figures S12–S14. Higher solution concentrations likely provide a larger number of PAc molecules available to interact with the perovskite, promoting the formation and stabilization of the desired α structure. However, slight differences in the phase formation are observed for PAc molecules with different ligands.

In addition to the phase stabilization as a function of PAc molecule type and concentration, we observed that the substrate treatment also affects the thickness of the deposited FAPbI_3_ films. Figure 4d demonstrates the variation in the thickness of FAPbI_3_ films deposited on the substrates treated with different PAc molecules and corresponding to different concentrations. In the films deposited on either bare FTO or on substrates washed with ethanol (dominated by δ phases), the thickness of the layer measured by profilometry is the same as that predicted by the QCM near the substrates (QCM1 in Figure 1a). However, as the content of α-FAPbI_3_ increases, the actual thickness of the film also deviates from the thickness predicted by the QCM, almost doubling in value corresponding to pure α-FAPbI_3_ film deposition. Furthermore, for PPAc treatment at higher concentrations, the XRD exhibits the formation of unknown lower dimensional phases (Figure S14) accompanied by an increase of three times in the measured thickness of the layer when compared to the predicted by the QCM. It is worth noting that all the samples were deposited on the same evaporation run and that the remarkable change in the thickness cannot be explained solely by the density differences between different crystalline structures. This suggests that the sticking coefficient of the precursor materials is also affected by the PAc molecule treatment, possibly also affecting the layer stoichiometry.

**Figure 5:**
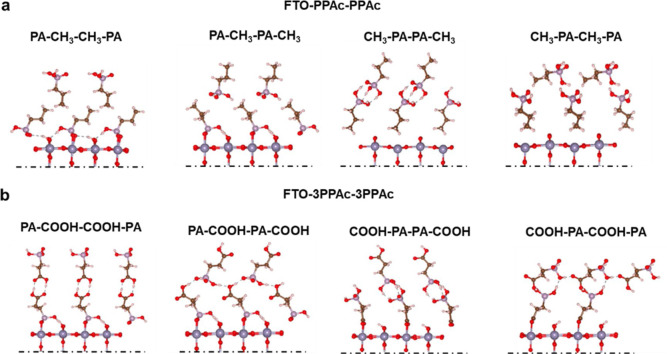
Ball and stick models of the bilayer of PAc molecules functionalizing the FTO surface. (a) FTO–PPAc–PPAc binding modes. (b) FTO–3PPAc–3PPAc binding modes. For clarity, only the top layer of FTO is shown.

Analyzing the above results points to important aspects of the growth mechanism, offering connections between our experimental and theoretical results. The experiments discussed in Figure 4 show that the mere presence of the monolayer passivating the substrate is insufficient to form α-FAPbI_3_. The data also confirm that having an excess of PPAc is essential for the formation of the α-FAPbI_3_ phase. However, it is observed that an excess of 3PPAc molecules leads exclusively to the formation of hexagonal FAPbI_3_ phases. These results suggest that the ligand (propyl vs carboxylic groups) also plays an important role in the α-FAPbI_3_ formation. Thus, we performed DFT calculations and studied ligand–ligand interactions to understand the role of the ligand on their migration during perovskite deposition. Here, we considered a bilayer arrangements of PPAc and 3PPAc molecules. Our models, depicted in Figure 5, encompass all the major modes of binding for which we calculated the BE according to eq S6 and reported in Table 2. We show the formation of H-bond pairs between the carboxyl groups of the top and bottom layers in the PA–COOH–COOH–PA configuration. We also observed a chain of H-bonds between the carboxyl and the PA groups of the top and bottom layers in the PA–COOH–PA–COOH ligand–ligand binding mode. These results are consistent with the large ligand–ligand BEs reported in Table 2. Conversely, for PPAc and its favorable mode of binding to the substrate (via PA), a weak interaction (−0.34 eV) between the top- and bottom-layer methyl groups was calculated (Table 2). These findings emphasize the significance of the ligand in PAc molecules, explaining why molecules with aliphatic moieties such as PPAc and EPAc are more effective in promoting the growth of α-FAPbI_3_, as their weaker ligand–ligand interactions can facilitate their migration to the top of the film during growth.

**Table 2:** BEs of the Upper-Layer PAc Molecule to the FTO-Bound Monolayer Corresponding to Various Intermolecular Binding Modes^a^

binding mode	PA–CH_3_–CH_3_–PA	PA–CH_3_–PA–CH_3_	CH_3_–PA–PA–CH_3_	CH_3_–PA–CH_3_–PA
BE (eV)	–0.34	–0.53	–1.60	–1.40
binding mode	PA–COOH–COOH–PA	PA–COOH–PA–COOH	COOH–PA–PA–COOH	COOH–PA–COOH–PA
BE (eV)	–1.02	–1.03	–2.70	–2.92

a BEs were calculated according to eq S5, and the definitions of different substrate–PAc and PAc–PAc binding modes are given in the caption of Figure 5.

### Phosphonic Acid Migration and the Sorption Rate of FAI

**Figure 6:**
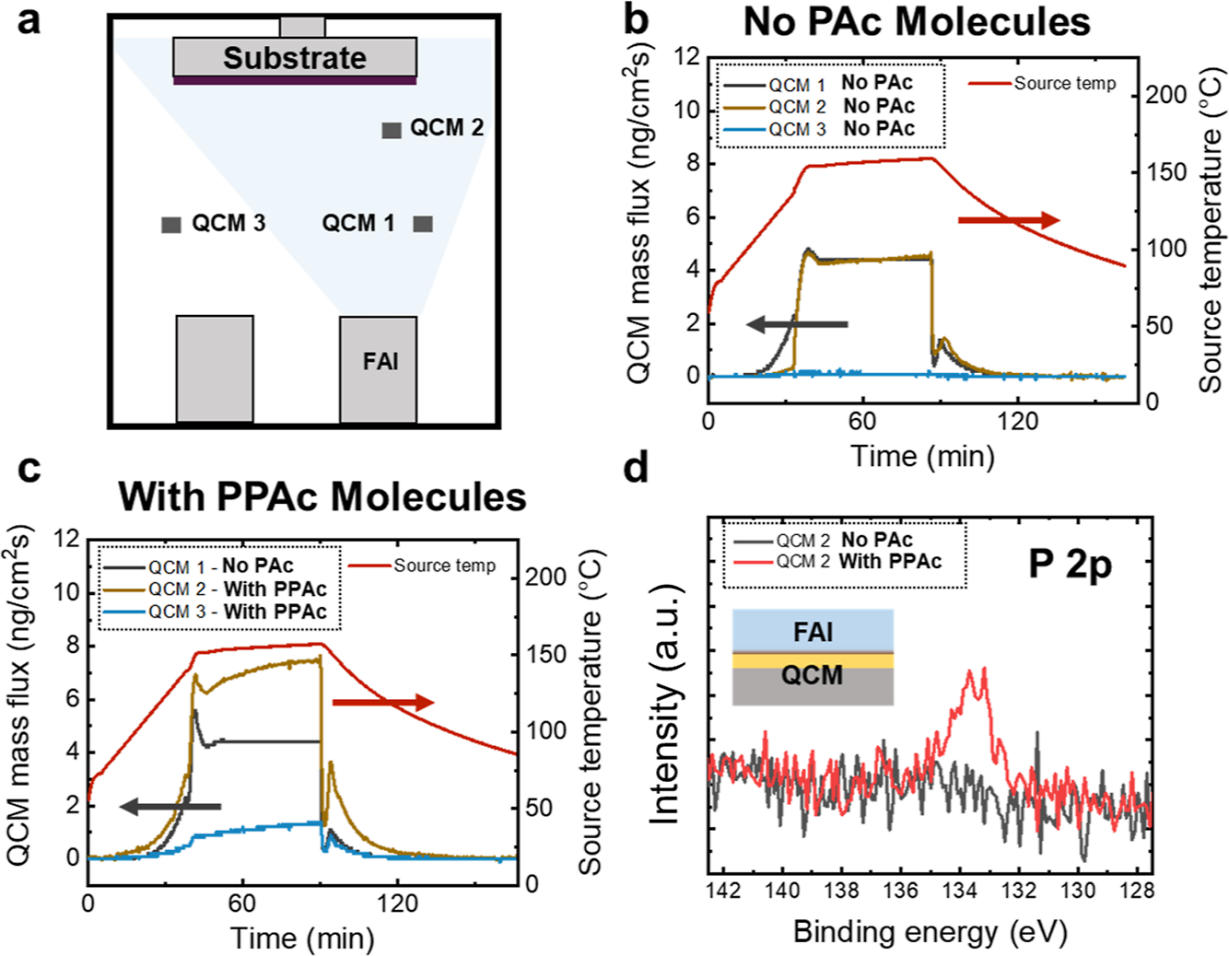
Deposition of FAI on bare and treated QCMs. (a) Schematic of the experimental setup. QCM mass flux for samples without treatment (b) and with PPAc treatment (c). (d) XPS P 2p scan measurements of QCMs with and without PPAc treatment after deposition of 200 nm of FAI.

To understand the effect of PAc molecules on FAI adsorption, we evaporated FAI thin films without the PbI_2_ (Figure 6a). The evaporation rate was kept constant between experiments by using a QCM without treatment near the FAI source, as shown in Figure 6b. We observe that treatment with the 3 mM PPAc (QCM 2) caused an increase in the mass flux of more than 50% with respect to the bare QCM 1 (Figure 6c). Furthermore, the treated QCM outside of the evaporation cone (QCM 3) also registered a significant mass flux, which might be related to the sorption of FAI itself or byproducts of the evaporated FAI. These findings confirm that the presence of excess PPAc molecules leads to increased FAI sorption, which, in turn, will affect the stoichiometry of the perovskite films. It is important to note that the increase in mass flux from FAI evaporation detected with the treated QCM not only occurs at the beginning of the evaporation but continues throughout the entire process. XPS analysis of the 200 nm FAI film on treated QCMs is shown in Figure 6d and an extended discussion of the perovskite stoichiometry deposited on treated and bare FTO substrates can be found in Figure S15 in the Supporting Information. The surface chemistry analysis revealed the presence of phosphorus (P 2p) on the surface of the FAI films. This may explain why we see increased sorption of FAI in the presence of PPAc treatment as the PPAc molecules appear to be migrating as we deposit FAI. This migration can be rationalized through the discussion in Figure 5 and Table 2, where we calculate weaker interactions between the ligands in PPAc. Figure S16 shows a comparison of the XPS P 2p signal for FAI films evaporated on gold-plated silicon substrates treated with different PAc molecules. The PAc molecules that are less effective in fostering the α-FAPbI_3_ growth also exhibited a lower intensity of the P 2p signal coming from the surface of the FAI layer. These findings suggest that the ligands of PPAc influence the migration of the molecules toward the surface. In turn, the FAI absorption efficiency may lead to the formation of stoichiometric perovskites and α-FAPbI_3_, which explains the increased N/Pb ratio observed in the XPS measurements (Figure S15). Moreover, it explains why performing an ethanol wash of the treated substrate (which removes excess ligands) results in obtaining only hexagonal phases.

### Proposed Mechanism for the Formation of α-FAPbI_3_

**Figure 7:**
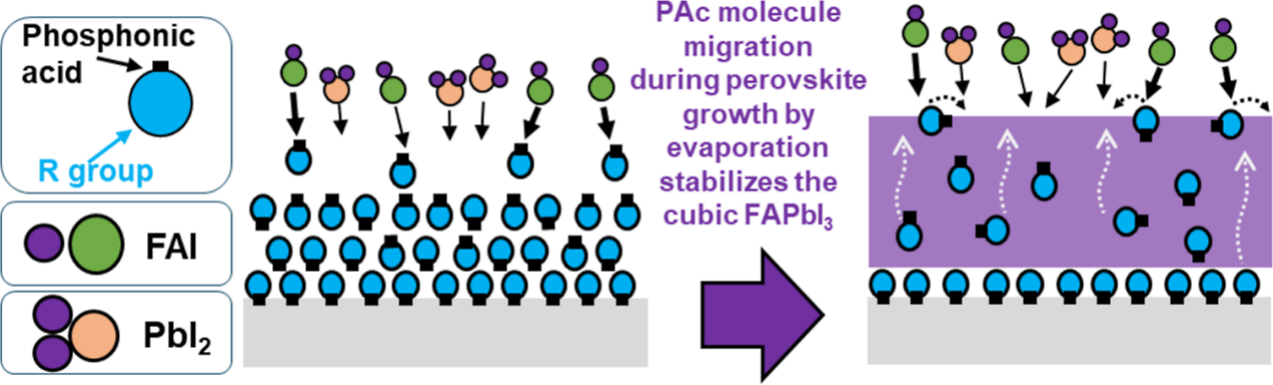
Proposed mechanism for the growth of α-FAPbI_3_ on phosphonic acid-treated substrates.

We propose a mechanism of α-FAPbI_3_ growth on the substrates treated with PPAc molecules as shown in Figure 7. First, the treatment with the PAc solution enables the adsorption of phosphonic acids onto the FTO and formation of P–O covalent bonds that results in the formation of a PPAc monolayer on the substrate. Excess PPAc molecules that exhibit weak interligand (e.g., propyl) interactions are able to migrate away from the substrate and interact with the PbI_2_ and FAI, where the phosphonic acid enables the stabilization of α-FAPbI_3_ during growth. This continued process increases the FAI sorption rate and enables the formation of the α-FAPbI_3_ through the decrease of the α phase growth energy, thus making it the favored phase to grow.

### Stability under Nitrogen and Humid Air

**Figure 8:**
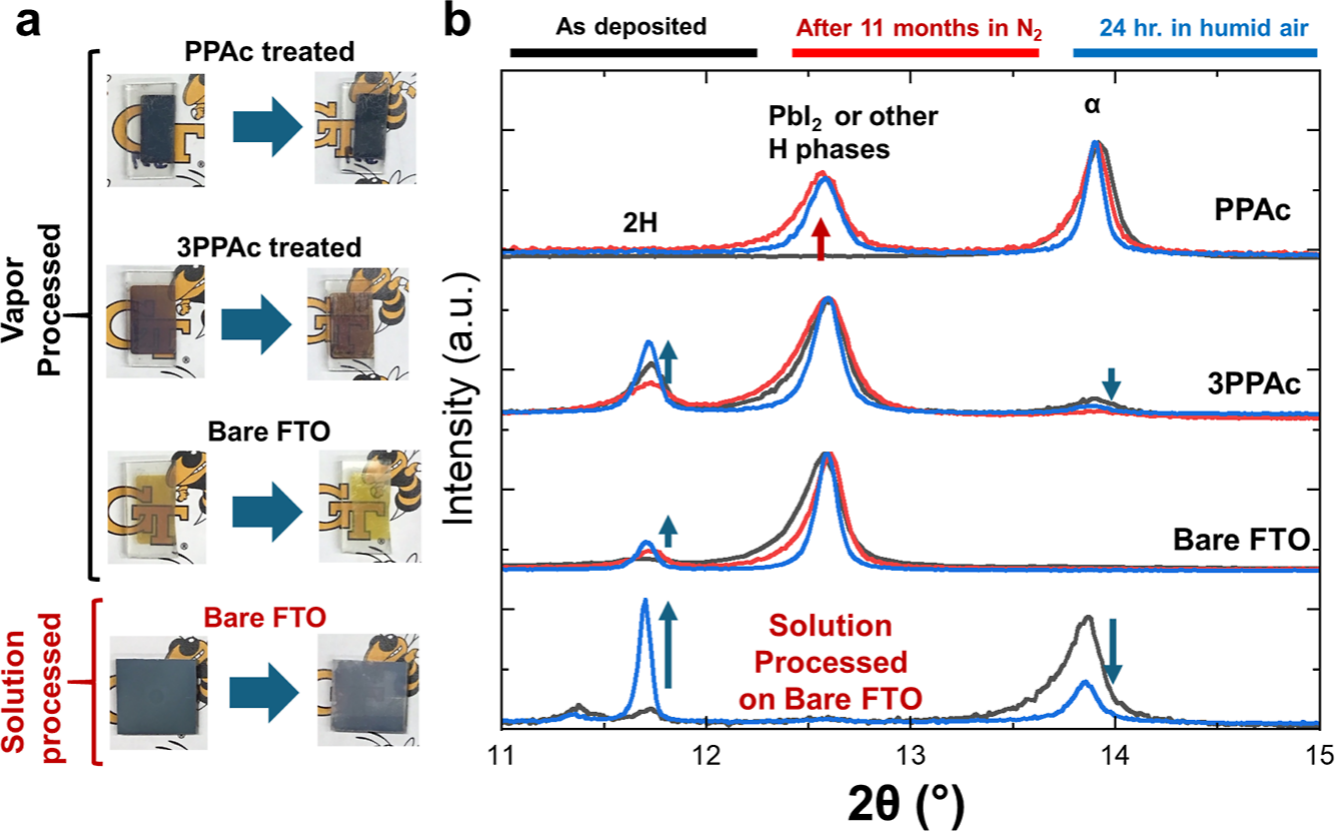
Stability test under nitrogen and humid air. (a) Photographs of thin films on FTO glass before and after exposure to saturated humid air (∼80% relative humidity). (b) XRD patterns of the film as deposited, after 11 months in a N_2_ box, and after 24 h of exposure to saturated humid air.

We measured the stability of the FAPbI_3_ films deposited via evaporation with and without PAc molecules and compared them with films deposited via spin-coating, as the α-FAPbI_3_ phase undergoes phase transformations in the presence of oxygen and moisture.^36–,38^ The photographs of the films show visual degradation after exposure to moist air over 24 h (Figure 8a), for all samples except the PPAc-treated materials. The PPAc-treated materials retain a significant amount of α-FAPbI_3_ even after an extended storage period of 11 months in a nitrogen glovebox (Figure 8b). The XRD analysis reveals the formation of some amount of PbI_2_ or 4H/6H hexagonal phases for the PPAc thin films, without any 2H-FAPbI_3_ formation. The films deposited on bare FTO and 3PPAc exhibit peaks that belong to the 2H-FAPbI_3_ and PbI_2_ or 4H/6H hexagonal phases. Figure S17 presents the complete XRD patterns. Importantly, exposure to humid air (∼80% relative humidity) for 24 h presents no major changes in the crystalline structure of the PPAc-treated samples. In contrast, solution-processed FAPbI_3_ films experience rapid degradation in the presence of moisture, with 2H-FAPbI_3_ becoming the predominant phase after 24 h. These results show that the presence of PPAc in the film not only promotes the growth of α-FAPbI_3_ but also prevents the phase transformation of α-FAPbI_3_ to the 2H phase.

## Conclusions

Substrate treatment with PAc molecules was successfully implemented to induce the growth of metal halide cubic perovskites, the α-FAPI_3_ phase, by thermal evaporation at room temperature. We investigated the role of substrate treatment on the phase purity and composition of the perovskite layer. The presence of the PAc group in the molecules was found to be most effective in promoting the α-FAPI_3_ phase formation by decreasing the growth energy associated with it to the greatest extent, as demonstrated by our DFT calculations. Notably, our findings revealed that the primary mechanism of driving the α-FAPI_3_ phase growth extends beyond the buried substrate–ligand interface. It is evident that unbound excess ligands play a crucial role in the α-FAPI_3_ phase growth by migrating toward the surface and enhancing the sorption rate of the organic perovskite precursor onto the substrate. This is supported by our XPS analysis and intermolecular BE calculations explaining the inability of PAc molecules with higher ligand–ligand BEs to promote the α phase growth. In addition, the PPAc-treated samples maintained the α-FAPI_3_ phase after 24 h of exposure to moist air. Our work underscores the importance of surface treatment through PAc molecular design to facilitate the α-FAPI_3_ phase growth for next-generation photovoltaic and light-emitting devices.

## Experimental Section

### Substrate Cleaning

The FTO substrates underwent a cleaning procedure involving sequential sonication for 15 min each in 2% mucasol (Schülke) solution, distilled water, acetone (Sigma-Aldrich, ≥99.5%), and isopropyl alcohol (Fischer Chemical). Subsequently, the substrates were dried using a nitrogen gun. Prior to the phosphonic acid treatment step, the substrates were exposed to a UV–ozone cleaner for 15 min.

### Phosphonic Acid Treatment

A solution of the phosphonic acid in ethanol (Sigma-Aldrich, 99%) was prepared and stirred for 1 h in a vortex mixer inside the glovebox. The concentration of the PAc solution varied from 1 to 10 mM. Subsequently, 100 μL of solution was dispensed onto the substrate and evenly spread. After allowing 1 min for settling, the spin-coating process was initiated, using a speed of 3000 rpm for 30 s with 1000 rpm/s of acceleration. Following the spin-coating step, the sample underwent annealing at 100 °C for 10 min.

### FAPbI_3_ Evaporation

Thermal evaporation was conducted using a Kurt J. Lesker MiniSpectros series low-temperature evaporator. Prior to deposition, the PbI_2_ and FAI deposition rates were accurately calibrated using dedicated QCMs positioned near the respective sources. The final thickness of the deposited materials on the substrate was continually monitored using another QCM placed near the substrate holder. During the evaporation process, the deposition rate of PbI_2_ was maintained at 0.4 Å/s, while the deposition rate of FAI was set to 0.44 Å/s. Typically, the evaporation was terminated when achieving a total thickness of around 400 nm on the substrates. To enhance film uniformity, the samples were rotated at a speed of 10 rpm throughout the evaporation procedure.

### Computational Methods

Bare and PAc-passivated stoichiometric slabs (with either PbI_2_ or FAI terminated surfaces) of α- and δ (2Η)–FAPbI_3_ were constructed from 2 × 2 × 4 and 1 × 1 × 3 supercells of their corresponding conventional unit cells, composed of 192 and 72 atoms, respectively (See Figures S9 and S10 where only the top few layers of reconstructed FAI- and PbI_2_-terminated slabs are shown, for clarity). DFT calculations with the Perdew–Burke–Ernzerhof exchange–correlation functional revised for solids and surfaces^39^ (revPBE) were performed using the Quantum Espresso suite of codes.^40^ Ultrasoft pseudopotentials were used to describe the interaction between the valence electrons and the ionic cores. Kohn–Sham orbitals were expanded in a plane-wave basis set with a kinetic-energy cutoff of 60 Ry and a density cutoff of 420 Ry. van der Waals interactions were incorporated via the empirical D3 dispersion correction scheme.^41^*E*_form_ of bulk FAPbI_3_ in δ and α phases were calculated based on the reaction FAI + PbI_2_ → FAPbI_3_, per eq S1, where *E*_FAPI_, , and *E*_FAI_ represent the DFT energies FAPbI_3_ and the precursors (PbI_2_ and FAI). The growth energy (*E*_g_, eq S4) defined as the summation of (a) the formation energy of the unit volume of FAPI (*E*_form_, eq S1) multiplied by the thickness (*h*), (b) the separation energy (*E*_sep_, eq S2), which is the energy required to cleave the slab from the bulk, and (c) the slab-ligand capping energy (*E*_cap_, eq S3) gained due to capping the slab with ligands, was computed for each passivated slab. In this formulation, *E*_sep_ is the energy required to form the unit area of the slab from the bulk phase, *E*_slab_ and *E*_bulk_ are total energies of the slab and the bulk phase, and A is the surface area of the slab. The capping energy (*E*_cap_) is also obtained from the total energies of the bare (*E*_slab_) and ligand-capped slabs (*E*_slab–PAc_). Furthermore, to study the FTO-treatment via PAc ligands, we constructed stoichiometric slab models of undoped SnO_2_ (110), which is thermodynamically most stable and will refer to it as FTO for consistency. FTO–PAc BEs were calculated from the DFT energies of the bare (*E*_FTO_) and PAc-capped (*E*_FTO–PAc_) slabs according to eq S5. To assess the ligand–ligand bond dissociation during the evaporative deposition of FAPbI_3_, we computed the ligand–ligand BE (BE_PAc–PAc_) per eq S6, from the total energies of the bilayer- and monolayer-treated FTO (*E*_(FTO–PAc–PAc)_ and *E*_(FTO–PAc)_) and the upper-layer ligand (*E*_PAc_).

### Characterization

XPS was conducted with a Thermo Scientific K-Alpha system using a monochromatic Al Kα X-ray source (1486.6 eV) with a 60° incident angle and a 0° photoemission angle, both measured from the sample normal. Survey and high-resolution scans were collected. High-resolution scans were taken with a 0.100 eV step size for P 2p, C 1s, N 1s, 13d, O 1s, Pb 4f, and Br 3d. XRD measurements were done in a third-generation Panalytical Empyrean diffractometer. GIWAXS was performed at the beamline 11-BM at the National Synchrotron Light Source II in Brookhaven National Laboratory. The samples were measured with incidence angles between 0.1 and 0.5° with a 20 s exposure time, using a beam with an energy of 13.5 keV, 0.2 mm × 0.05 mm size, 1 mrad divergence, and an energy resolution of 0.7%. UV–vis absorption spectra were taken with a Cary 5000 UV–vis/NIR spectrometer. The thickness of the films was measured with a Bruker Dektak XT profilometer. CL spectra were taken at the Center for Nanophase Materials Science at Oak Ridge National Laboratory with a FEI Quattro environmental SEM and Delmic Sparc CL collection module. Electron beam energy was 5 keV and current was 7 pA. CL signal acquisition time was 100 ms with a pixel size of 40 nm. The data were processed using python libraries developed by Sergei Kalinin and Jonghee Yang.^32^

## Supporting Information Available

Additional information related to this article, including complementary figures and discussion, XRD diffraction patterns, XPS data, UV–vis absorption spectra, and further details on the theoretical calculations (PDF)
