# Supplementary material for: Tailoring Interface Energies via Phosphonic Acids to Grow and Stabilize Cubic FAPbI3 Deposited by Thermal Evaporation
Source: J Am Chem Soc. 2024 Jun 27;146(27):18459–69. doi: 10.1021/jacs.4c03911 (PMC11240563; doi:10.1021/jacs.4c03911)
Supplement: Supplementary file 1 — ja4c03911_si_001.pdf [file ja4c03911_si_001.pdf]

Supporting information

Tailoring Interface Energies via Phosphonic Acids to Grow and Stabilize Cubic $FAPI_3$ Deposited by Thermal Evaporation

Andrés-Felipe Castro-Méndez[1], Farzaneh Jahanbakhshi[2], Diana K. LaFollette[1], Benjamin J. Lawrie[3,4], Ruipeng Li[5], Carlo A. R. Perini[1], Andrew M. Rappe[2], Juan-Pablo Correa-Baena[1,6*]

[1]School of Materials Science and Engineering, Georgia Institute of Technology, North Ave NW, Atlanta, Georgia 30332, USA

[2]Department of Chemistry, University of Pennsylvania, Philadelphia, PA 19104-6323, USA

[3]The Center for Nanophase Materials Sciences, Oak Ridge National Laboratory, Oak Ridge, TN, 37831, USA

[4]Materials Science and Technology Division, Oak Ridge National Laboratory, Oak Ridge, TN, 37831, USA

[5]National Synchrotron Light Source II (NSLS-II), Brookhaven National Laboratory, Upton, New York 11967, USA

[6]School of Chemistry and Biochemistry, Georgia Institute of Technology, North Ave NW, Atlanta, Georgia 30332, USA

Corresponding author: JPCB jpcorrea@gatech.edu

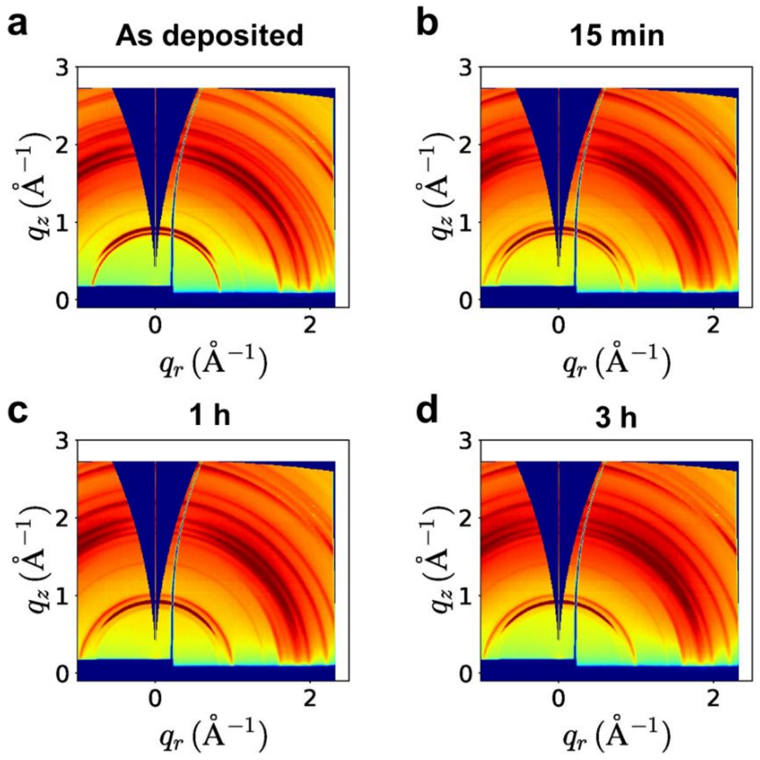

Figure S1. GIWAXS patterns of $FAPbI_3$ films on bare FTO annealed at 150 °C by varying time.

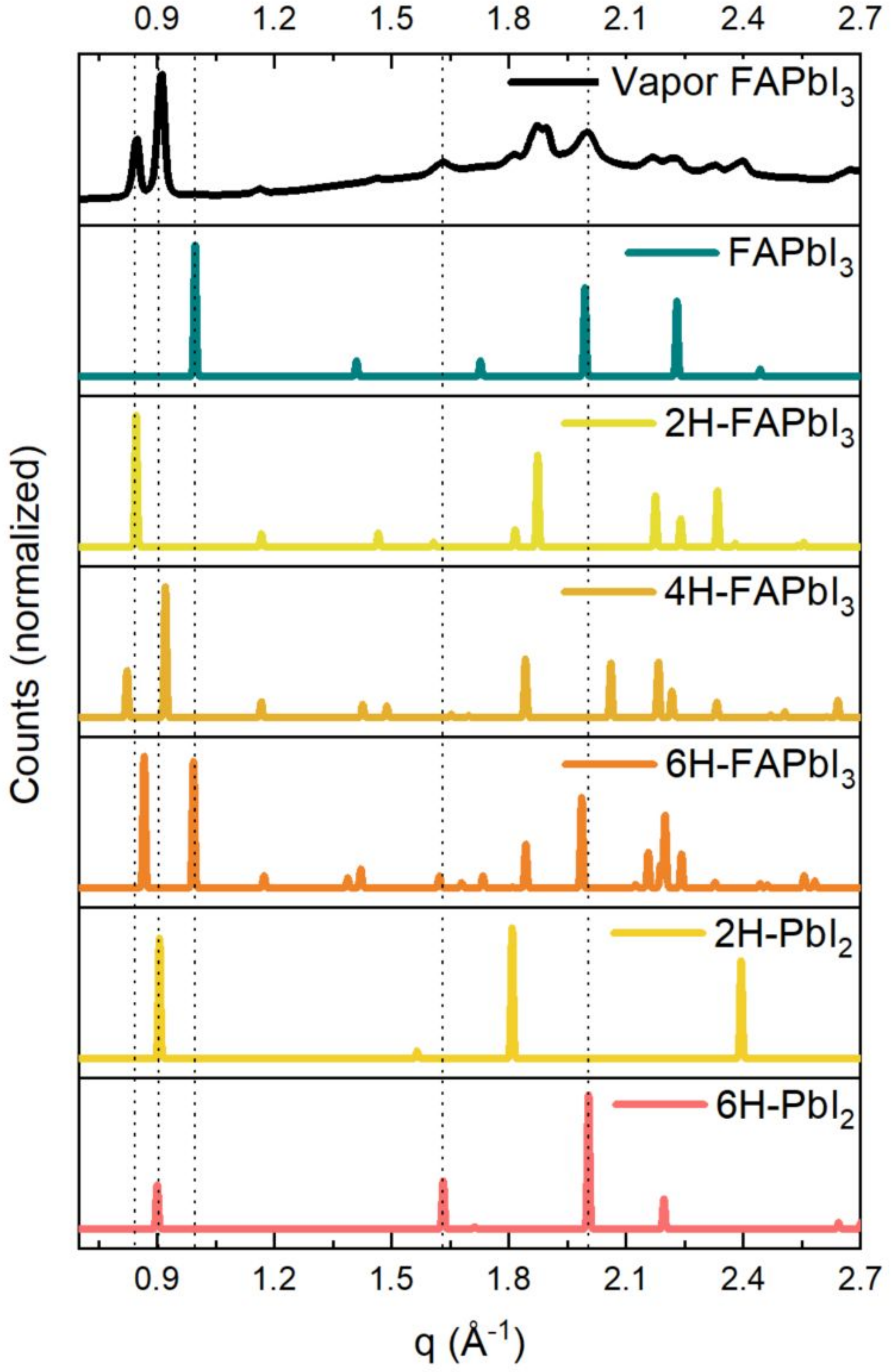

Figure S1. Circular integration of GIWAXS pattern of $FAPbI_3$ evaporated on bare FTO (black trace) and comparison with simulated patterns for the possible phases.

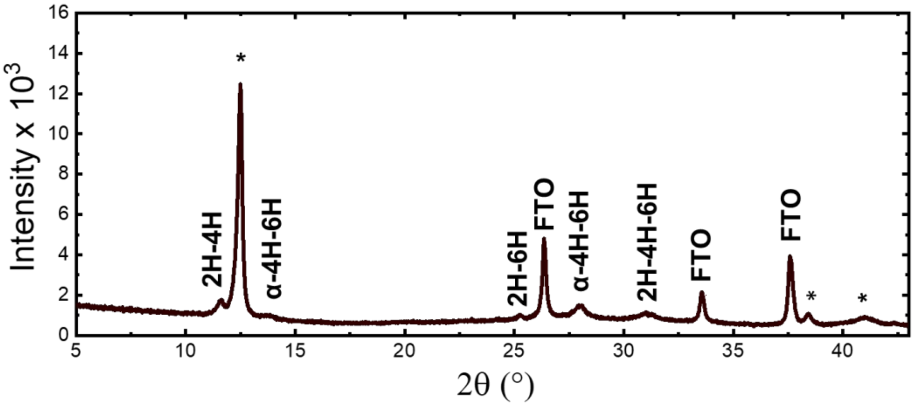

Figure S3. XRD pattern of as-deposited $FAPbI_3$ film of bare FTO

Figure S3. shows the X-ray diffraction (XRD) pattern of a film deposited on FTO, revealing the presence of the 2H $FAPbI_3$ polytype, as evidenced by the presence of the characteristic peak of the (100) plane at $2\theta = 11.65°$. Additionally, a small amount of the cubic phase, α-$FAPbI_3$, was detected with a peak observed at $2\theta = 13.9°$. However, a peak at $2\theta = 12.5°$ exhibited the highest intensity, suggesting that another non-perovskite phase constitutes the majority of the film. This peak could potentially be associated with either PbI_2 or other hexagonal polytypes, such as 4H and 6H. Peaks from the 6H-PbI_2 phase match with the positions of the peaks marked with an *. However, precise phase identification is difficult in polycrystalline thin films due to the overlap of the peaks between all the phases and the low signal of the peaks at higher angles.

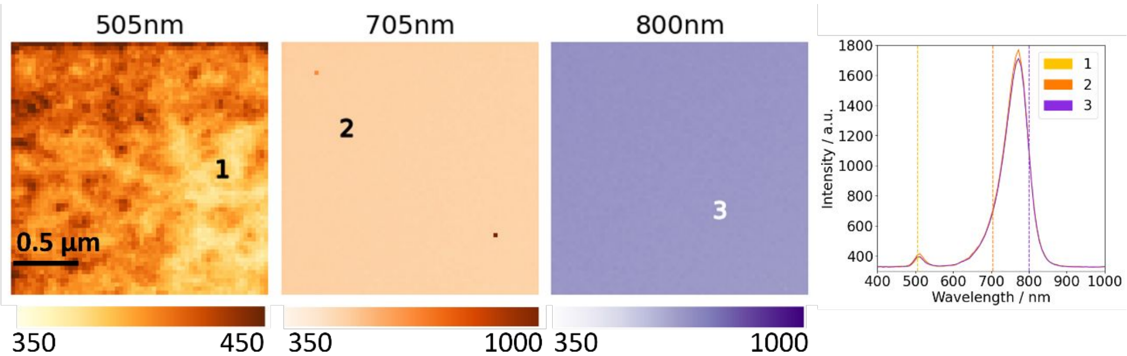

Figure S4. CL-SEM intensity maps and point CL spectra of co-evaporated films on bare FTO

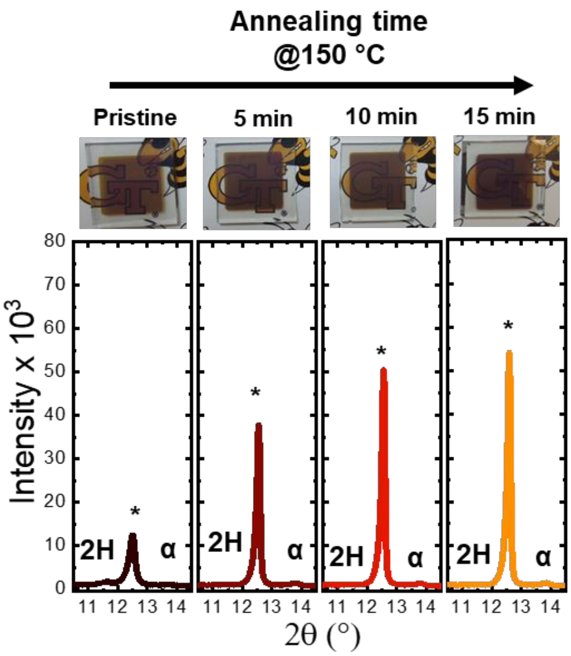

Figure S5. XRD patterns of $FAPbI_3$ films on bare FTO annealed by varying time. The *-marked peak is associated with hexagonal PbI_2 or $FAPbI_3$ polytypes.

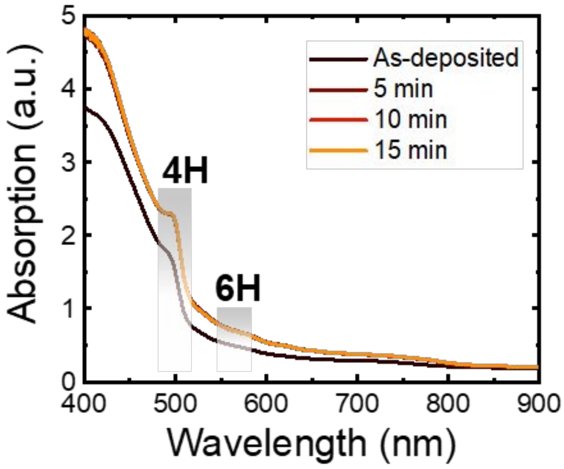

Figure S6. UV-VIS spectroscopy of $FAPbI_3$ films on bare FTO annealed by varying time.

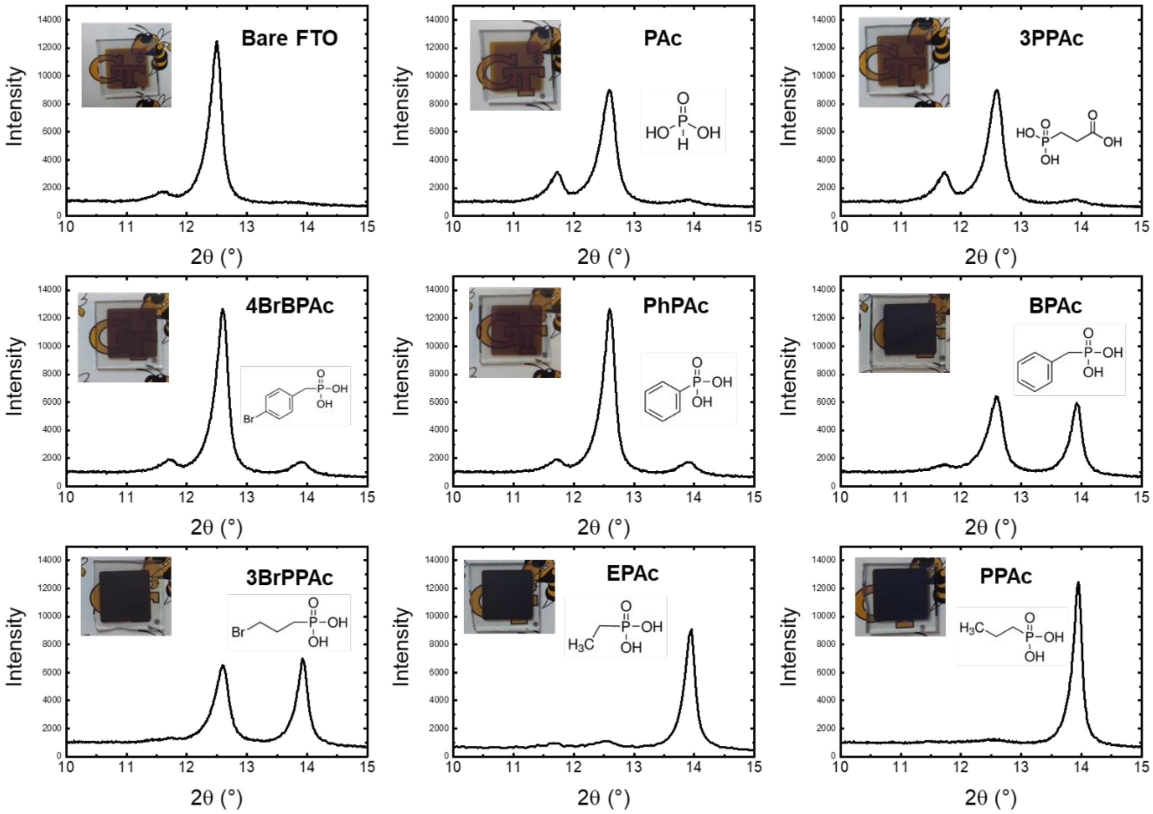

Figure S7. XRD patterns of $FAPbI_3$ films on FTO functionalized with different functional groups on the R-ligand of the phosphonic acid and concentration of 5mM. The peaks for PPAc and EPAc match closely those of the α-$FAPbI_3$ at around 2θ= 13.9° whereas the rest of the PAc molecules produce a combination of both α-$FAPbI_3$ and hexagonal phases, with peaks at 2θ= 12.5° and 11.6°.

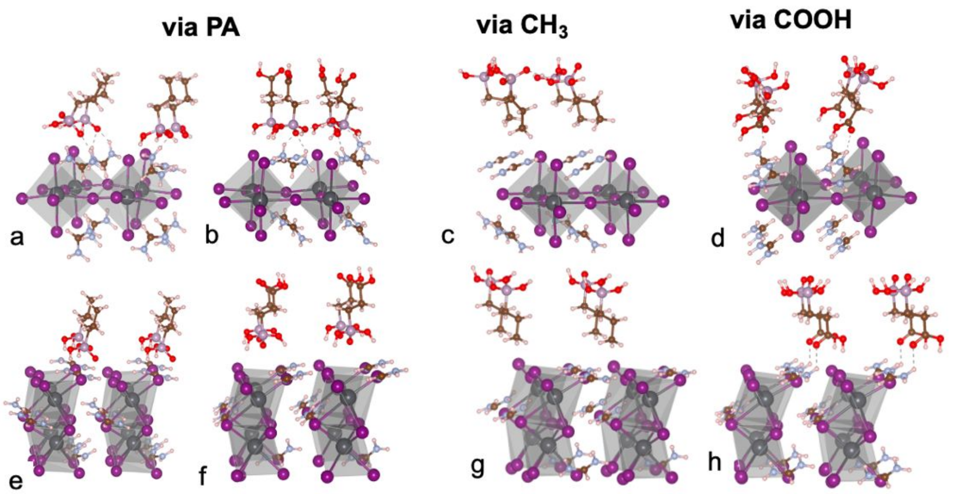

Figure S8. Proposed capping mechanism of $FAPbI_3$ via phosphonic acids. Ball and stick models of PPAc (a, c, e, g) and 3PPAc (b, d, f, h) phosphonic acids capping FAI-terminated slabs of α-(upper panel) and 2H-$FAPbI_3$ (lower panel) both via PA (a, b, c, d), CH_3 of PPAc (e, g) and COOH of 3PPAc (f, h) are shown. Dashed lines indicate H-bonds between H of terminal FA^+ and O of phosphonate (a, b) as well as O of carboxyl group (d, h). O, C, N, H, P, Pb and I atoms are depicted as red, brown, light blue, white, lavender, grey and magenta spheres. For clarity, only the top few layers of the slabs are shown.

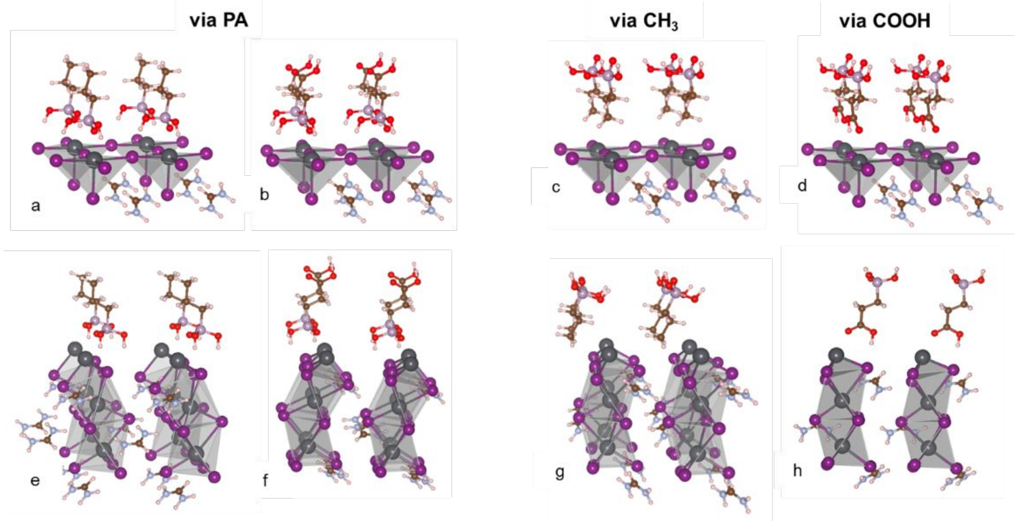

Figure S9. Proposed capping mechanism of $FAPbI_3$ via phosphonic acids. Ball and stick models of PPAc (a, c, e, g) and 3PPAc (b, d, f, h) phosphonic acids capping PbI_2-terminated slabs of α-(upper panel) and 2H- $FAPbI_3$ (lower panel) via PA (a, b, c, d), CH_3 of PPAc (e, g) and COOH of 3PPAc (f, h) are shown. O, C, N, H, P, Pb and I atoms are depicted as red, brown, light blue, white, lavender, grey and magenta spheres. For clarity, only the top few layers of the slabs are shown.

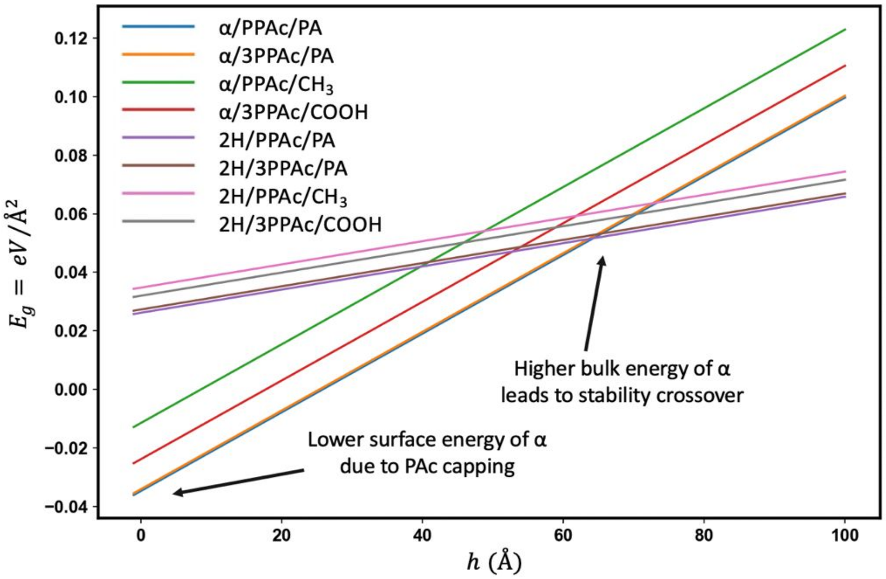

Figure S10. The growth energy, E_{g} (eV/Å^2), for PAc-capped PbI_2-terminated $FAPbI_3$ versus the grown thickness (h) obtained via eq(S4). The energy terms for each capped slab model are provided in Table 1.

$$E_{\mathrm{form}} = E_{\mathrm{FAPI}} - E_{\mathrm{FAI}} - E_{\mathrm{PbI_2}} \quad \text{eq(S1)}$$

$$E_{\mathrm{sep}} = (E_{\mathrm{slab}} - E_{\mathrm{bulk}})/A \quad \text{eq(S2)}$$

$$E_{\mathrm{cap}} = (E_{\mathrm{slab-PAc}} - E_{\mathrm{slab}} - E_{\mathrm{PAc}})/A \quad \text{eq(S3)}$$

$$E_g = E_{\mathrm{form}}.h + E_{\mathrm{sep}} + E_{\mathrm{cap}} \quad \text{eq(S4)}$$

$$\mathrm{BE_{PAc}} = E_{\mathrm{FTO-PAc}} - E_{\mathrm{PAc}} - E_{\mathrm{FTO}} \quad \text{eq(S5)}$$

$$\mathrm{BE_{PAc-PAc}} = E_{\mathrm{(FTO-PAc-PAc)}} - E_{\mathrm{(FTO-PAc)}} - E_{\mathrm{P}}\text{[illegible]} \quad \text{eq(S6)}$$

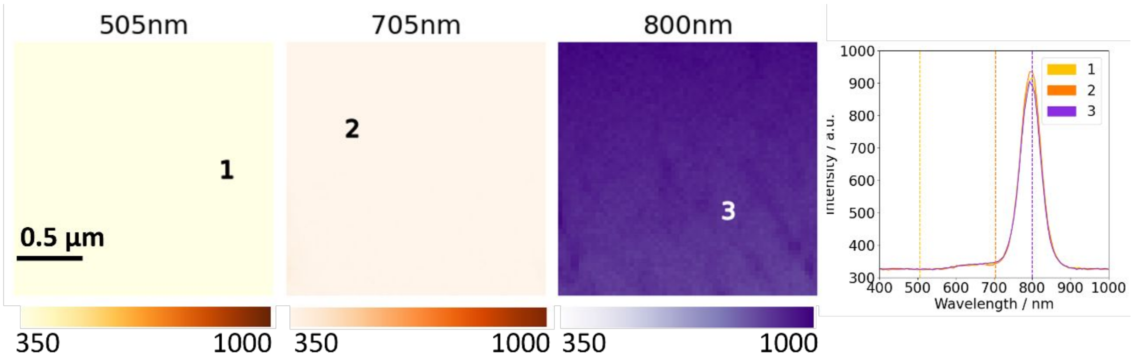

Figure S11. CL-SEM intensity maps and point CL spectra of co-evaporated films on 3mM PPac

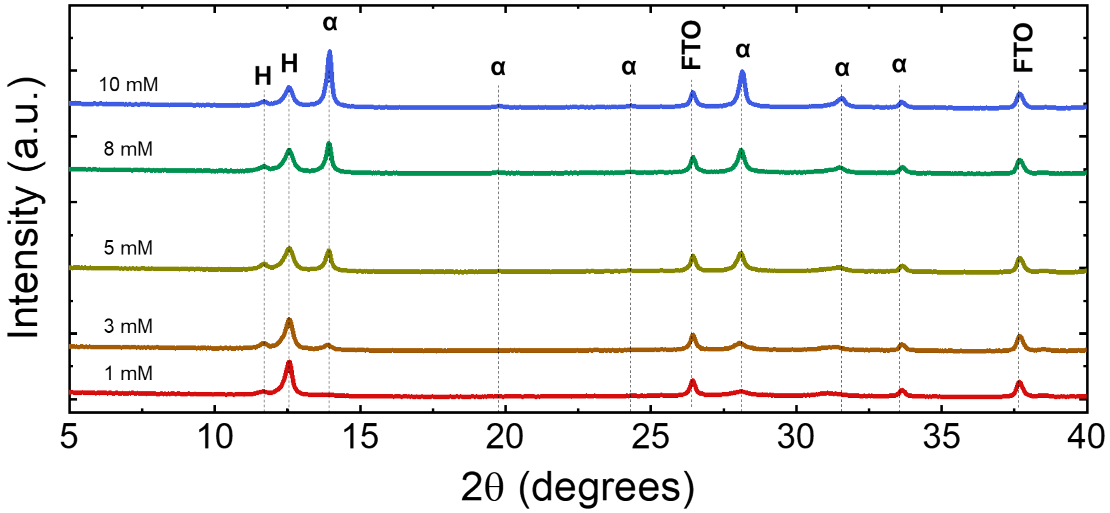

Figure S12. XRD patterns of $FAPbI_3$ films deposited on 3BrPPAc functionalized substrate with varying concentration. H = hexagonal phases.

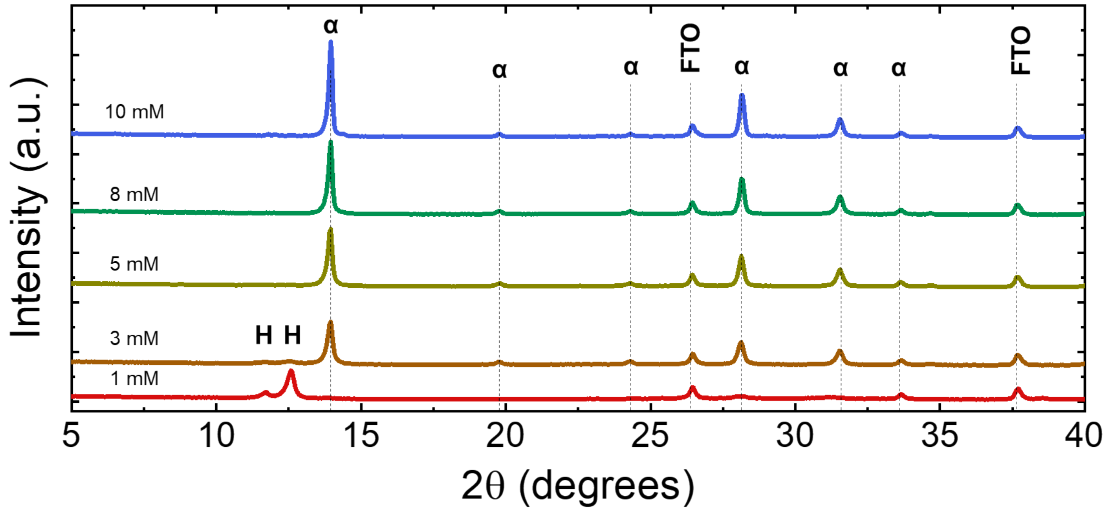

Figure S13. XRD patterns of $FAPbI_3$ films deposited on EPAc functionalized substrate with varying concentration.

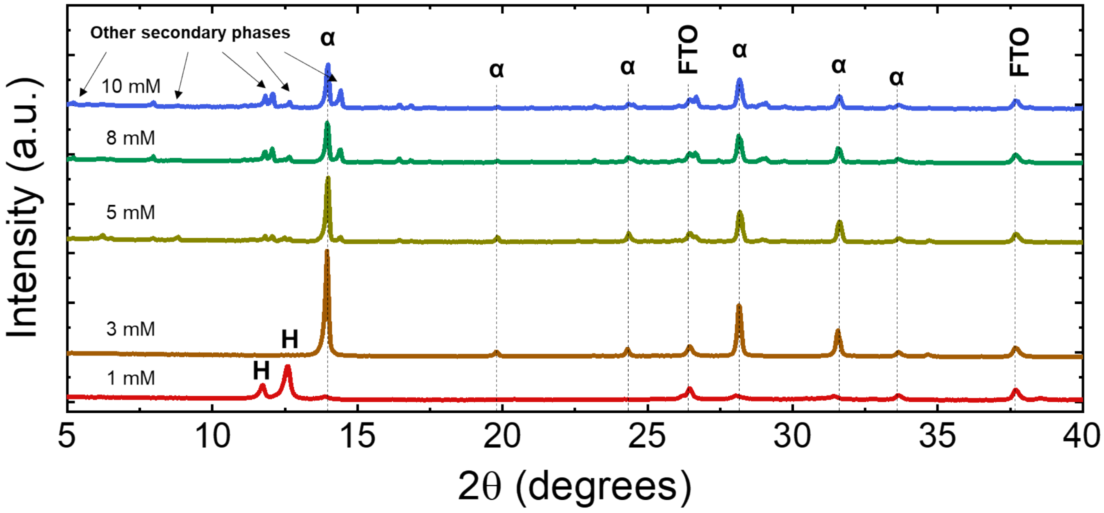

Figure S14. XRD patterns of $FAPbI_3$ films deposited on PPAc functionalized substrate with varying concentration.

Table S1. The energy corresponding to the first ligand binding the FTO surface, at PBEsol+D3 level of theory, for PA, CH_3 (of PPA) and COOH (of 3PPAc) binding modes.

**mode**	**PA**		**CH_3 (or COOH)**	
**PAc**	**PPAc**	**3PPAc**	**PPAc**	**3PPAc**
**BE_{PAc} (eV)**	-4.11	-3.99	-0.6	-1.51

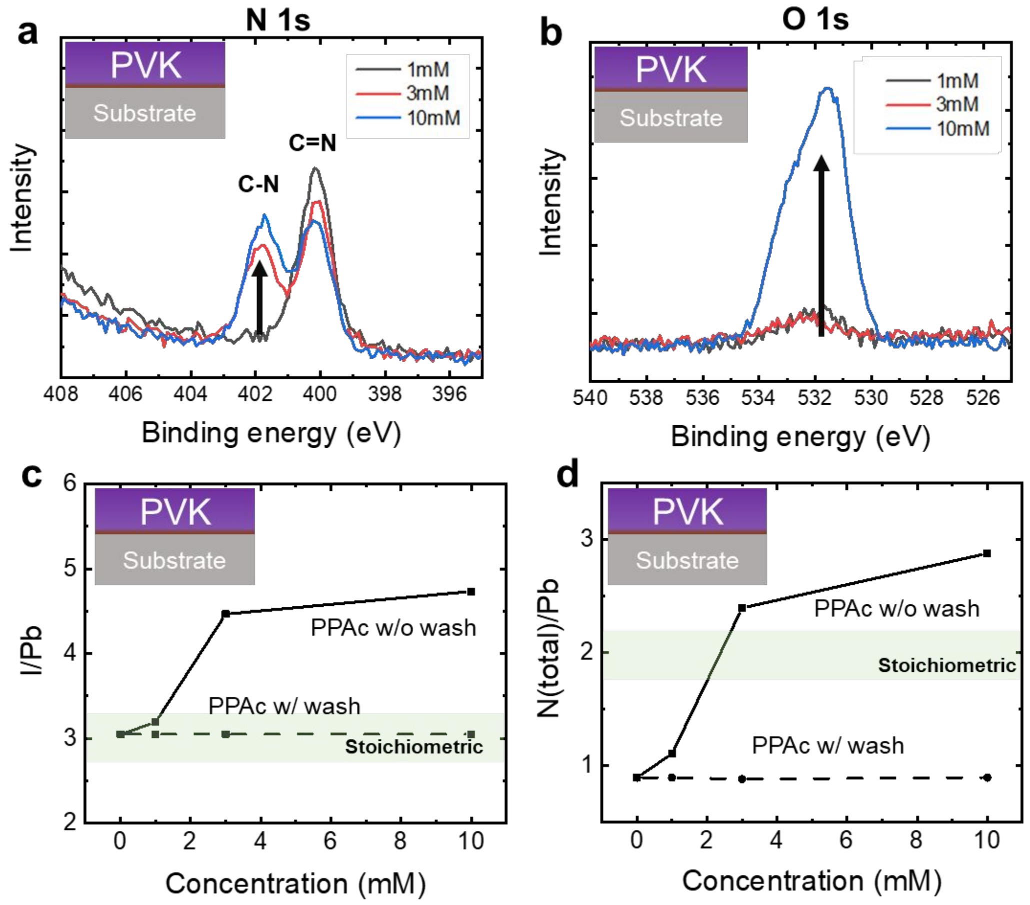

Figure S15. XPS analysis for Perovskite films deposited on substrates functionalized with different concentrations of PPAc molecules. (a) N 1s scan. (b) O 1s scan. (c) I/Pb elemental ratio. (d) N/Pb elemental ratio

Surface chemistry discussion of FAPbI3 and FAI films deposited on surface treated and bare FTO

To further understand the proposed mechanism of PPAc molecules migrating during thin film growth, we studied the surface chemistry of perovskite films grown on treated samples and bare substrates. In general, having a ratio of less than 3:1 for iodine:lead, and less than 1:1 for the organic:lead (often obtained from the nitrogen signal in XPS) is an indication of forming non-perovskite phases. In Figure S15, we present the ratio of I:Pb and N:Pb as a function of PAc concentration, using PPAc as an example, as it is the most effective molecule driving the conversion of the deposited $FAPbI_3$ film to the α phase. A stoichiometric α-$FAPbI_3$ is expected to have an I:Pb ratio of 3:1 and an N:Pb ratio of 2:1. The XPS data of films deposited on bare FTO reveals an I:Pb ratio around 3:1, as expected in a $FAPbI_3$ film, but a N:Pb ratio of about 0.9:1. This finding suggests that the films deposited on bare FTO have a deficiency of FA^+ cations. We speculate that this deficiency is contributes to the formation of the observed hexagonal phases. This is in addition to our findings that without capping $FAPbI_3$ with phosphonic acids the growth energy of the δ-$FAPbI_3$ is more favorable than that of the α-$FAPbI_3$ (Table 1). On the other hand,

as the concentration of PPAc increases, the ratio of N:Pb also increases, achieving a value close to 2 in films where only the α phase can be observed in the diffraction pattern. For concentrations above 5 mM of the PPAc molecule, we found ratios above 2:1 of N:Pb. This high level of organic cations correlates with the emergence of lower dimensional phases in the film observed in XRD (Figure S14). Moreover, the O 1s XPS signal increases as we increase the PPAc concentration (Figure S15), suggesting the adsorption of oxygen species as more FA, which is hygroscopic[38], is incorporated in the film. It is possible that this signal belongs to the oxygen species in the PAc molecules. In addition, we expect the excess of organics in $FAPbI_3$ films to induce the formation of low dimensional phases, analogously to what is observed in thermally evaporated $MAPbI_3$ perovskites[11]. Conversely, we observed no change in the I:Pb or N:Pb ratios of measured XPS for $FAPbI_3$ films deposited on the washed substrates, suggesting that a monolayer of PPAc is not enough to drive sufficient adsorption of FA molecules.

In addition to the FAI C=N bond peak signal (at 400 eV), the XPS N 1s scan shows the presence of a C-N bond peak (at 401.85 eV, Fig. S16a) in the films on substrates that are functionalized with PAc molecules. It is possible that this signal belongs to FAI byproducts with C-N bond characteristics and that are incorporated in the perovskite structure during deposition. However, no clear structural effects were shown as this XPS peak increases in intensity.

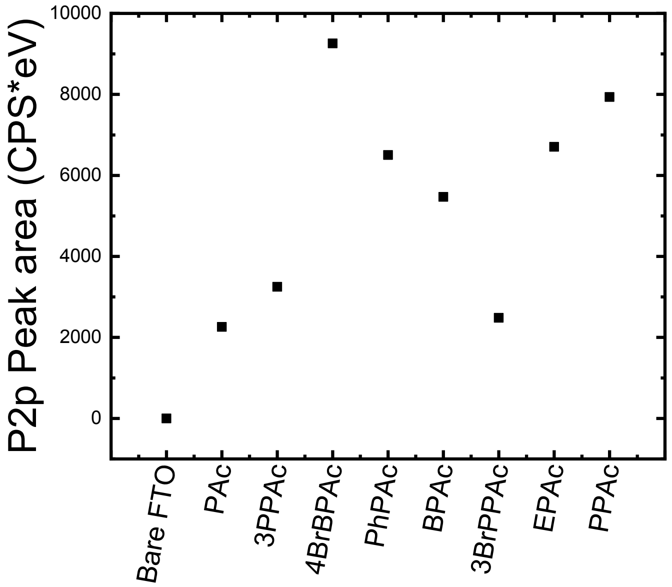

Figure S16. Integration of P 2p XPS peak on FAI thin films (200 nm) deposited via thermal evaporation on Si/Au/phosphonic acid substrates.

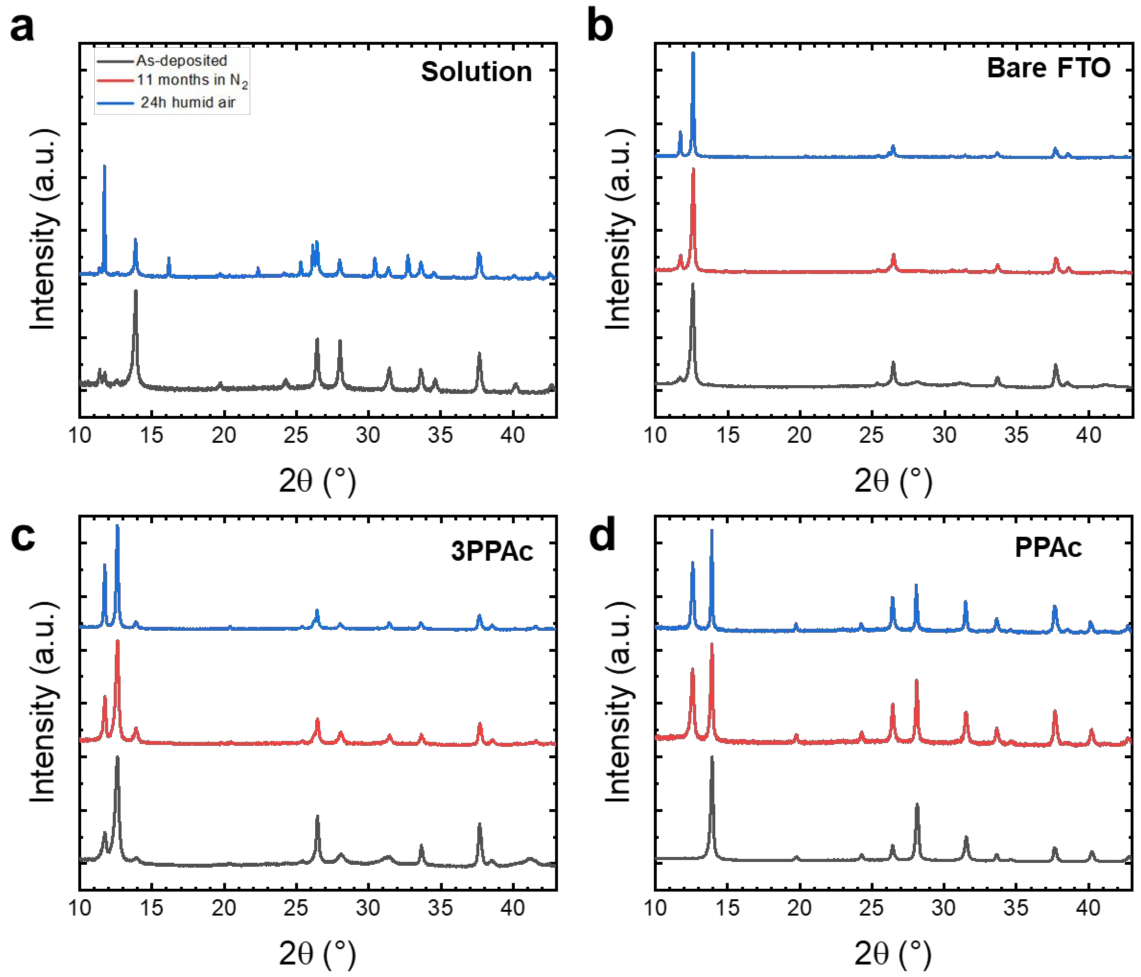

Figure S17. XRD patterns of FAPbI3 deposited by (a)spin-coating (Solution) and evaporation on (b) bare FTO, (c) 3PPAc treated FTO, and (d) PPAc treated FTO.
